# *Plasmodium berghei*-Released Factor, *Pb*TIP, Modulates the Host Innate Immune Responses

**DOI:** 10.3389/fimmu.2021.699887

**Published:** 2021-12-07

**Authors:** Inderjeet Kalia, Rajesh Anand, Afshana Quadiri, Shreya Bhattacharya, Bijayalaxmi Sahoo, Agam Prasad Singh

**Affiliations:** ^1^ Infectious Diseases Laboratory, National Institute of Immunology, New Delhi, India; ^2^ Department of Biological Sciences and Engineering, Maulana Azad National Institute of Technology, Bhopal, India

**Keywords:** immunomodulation, immune evasion, T cell immunomodulatory protein, malaria, macrophages altered phenotype, M2 macrophages, immune-tolerance

## Abstract

The *Plasmodium* parasite has to cross various immunological barriers for successful infection. Parasites have evolved mechanisms to evade host immune responses, which hugely contributes to the successful infection and transmission by parasites. One way in which a parasite evades immune surveillance is by expressing molecular mimics of the host molecules in order to manipulate the host responses. In this study, we report a *Plasmodium berghei* hypothetical protein, *Pb*TIP (PbANKA_124360.0), which is a *Plasmodium* homolog of the human T-cell immunomodulatory protein (TIP). The latter possesses immunomodulatory activities and suppressed the host immune responses in a mouse acute graft-*versus*-host disease (GvHD) model. The *Plasmodium berghei* protein, *Pb*TIP, is expressed on the merozoite surface and exported to the host erythrocyte surface upon infection. It is shed in the blood circulation by the activity of an uncharacterized membrane protease(s). The shed *Pb*TIP could be detected in the host serum during infection. Our results demonstrate that the shed *Pb*TIP exhibits binding on the surface of macrophages and reduces their inflammatory cytokine response while upregulating the anti-inflammatory cytokines such as TGF-β and IL-10. Such manipulated immune responses are observed in the later stage of malaria infection. *Pb*TIP induced Th2-type gene transcript changes in macrophages, hinting toward its potential to regulate the host immune responses against the parasite. Therefore, this study highlights the role of a *Plasmodium*-released protein, *Pb*TIP, in immune evasion using macrophages, which may represent the critical strategy of the parasite to successfully survive and thrive in its host. This study also indicates the human malaria parasite TIP as a potential diagnostic molecule that could be exploited in lateral flow-based immunochromatographic tests for malaria disease diagnosis.

## Introduction

Malaria is a debilitating infectious disease in vertebrates, including humans, and is transmitted through the bites of the *Plasmodium* carrier *Anopheles* mosquitoes. In 2019, approximately 229 million malaria cases were reported, with about 409,000 deaths, and these figures will likely shoot up due to the recent coronavirus outbreak, restricting mosquitoes and parasite control measures (1, 2).

Natural immunity against malaria is crucial to protect the host from the severe form of the disease and anemia (3). Natural immunity develops in a population of a malaria-endemic area by repeated *Plasmodium* exposure for many years through mosquito bites. However, it does not provide sterile protection against the disease; also, it is short lived and fades away quickly (3, 4). Immune responses against blood-stage parasites are complex and possibly initiated when parasite-derived glycosylphosphatidylinositol (GPI), DNA, and metabolic products (e.g., hemozoin and uric acid) are released during the blood stage of malaria infection (5–8). These parasite-derived products are sensed by Toll-like receptors (TLRs) and nucleotide-binding oligomerization domain (NOD)-like receptors containing pyrin domain 3 on dendritic cells (DCs) and macrophages and lead to the activation of CD4^+^ and CD8^+^ T cells, as well as parasite-specific antibodies to contain the blood-stage infection (9–11). However, observational studies have suggested that the human malaria parasite always persists for several weeks or months and that immunity to malaria develops slowly, indicating that the parasite may have developed strategies to suppress the host immune responses in order to thrive in the host (12). Previous studies have suggested that the malaria parasite can inhibit the activation of parasite-specific CD4^+^ T-cell responses by inducing the apoptosis of DCs, downregulating co-stimulatory molecules, and/or suppressing antigen presentation by DCs (13–15). The *Plasmodium* parasite also induces apoptosis and anergy of the activated CD4^+^ T and B cells (16–18). Observational studies in mice and humans have suggested the role of natural regulatory T cells (Tregs) in malaria infection and the presence of increased Tregs during the infection (19, 20). However, the mechanisms of host immune manipulation by the parasite remain poorly understood.

The manipulation of host immune pathways by parasites involving active discharge of molecules, analogous to host cytokines, has recently been reported during malaria infection (21). The *Plasmodium* parasite-released homolog of the mammalian migration inhibitory factor (MIF) induces antigen-experienced CD4^+^ T cells into short-lived effector cells rather than memory cells through binding on its CD74 receptor in DCs and macrophages during blood-stage malaria (22, 23). Such ability of pathogens to circumvent the host defense mechanisms is also evident in bacterial infection and chronic infection of filarial worms and schistosomes (24, 25). In nematodes, immune evasion strategies promote the survival of parasites within the host by altering the activation of the host immune responses during infection. The worm-secreted/excreted products such as serpins, miRNA, and proteases are capable of mimicking host molecules to carry out immune modulation in the host (25, 26). Various reports highlight such ability of the parasites to circumvent the host anti-parasitic responses, and this understanding is critical in designing therapeutic interventions.

Here, we report that the malaria parasite expresses a T-cell immunomodulatory protein (*Pb*TIP) on the surface of merozoites that is homologous to human T-cell immunomodulatory protein (TIP). TIP was shown to have a protective role in a mouse model of graft-*versus*-host disease (GvHD) by manipulating T-cell responses (27). The *Plasmodium berghei* homolog of TIP, *Pb*TIP, is exported on the infected erythrocyte surface upon infection by merozoites. Fragmentation of the surface-expressed *P. berghei Pb*TIP, likely by protease(s), resulted in its shedding from the red blood cell (RBC) surface into host circulation. Once in circulation, *Pb*TIP exhibited binding on host macrophages and reduced the expressions of their inflammatory cytokine genes while upregulating the expressions of the Th2-type/anti-inflammatory cytokine genes. *Pb*TIP also helps in parasite growth *in vivo*.

## Results

### A *Plasmodium* Hypothetical Protein Is Homologous to Human T-Cell Immunomodulatory Protein

**Figure 1:**
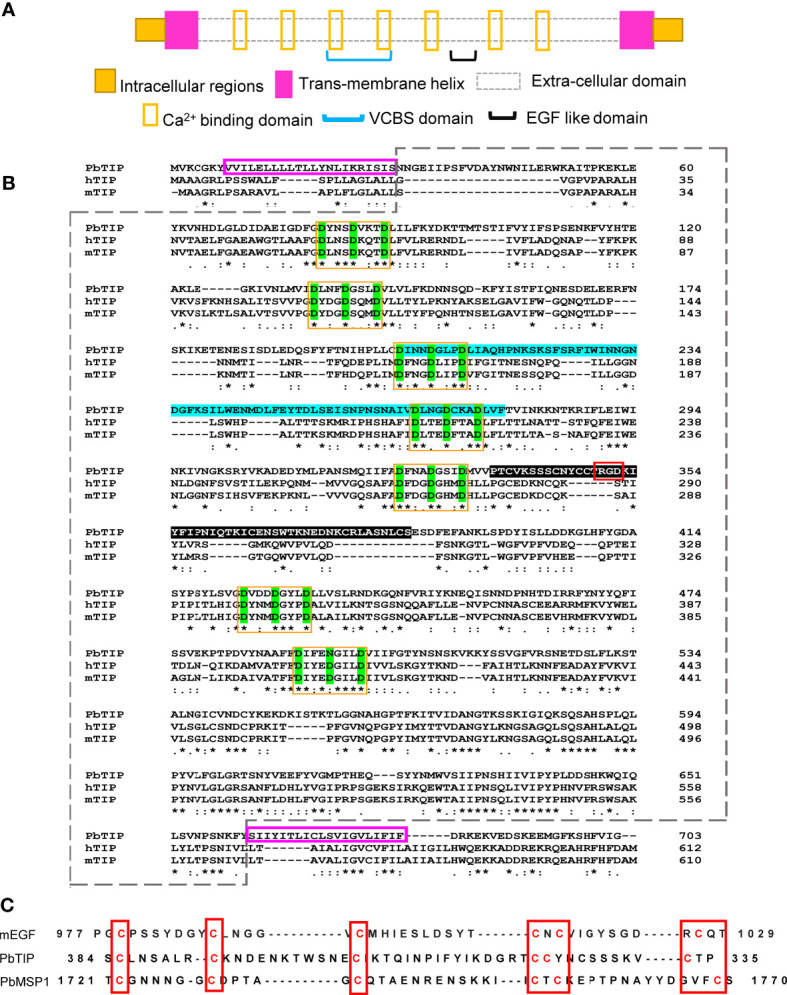
A hypothetical *Plasmodium* protein is homologous to human T-cell immunomodulatory protein (TIP) and is predicted to contain various putative adhesion domains. **(A)** Summary of the various putative domains in the *Plasmodium berghei* TIP. **(B)** The PbANKA_124360.0 encoded protein designated as *Pb*TIP shared 27.88% and 28.33% homology with human TIP and mouse TIP, respectively, along their lengths. *Pb*TIP is predicted to contain two transmembrane (TM) helices [7–29 and 662–681 amino acid (AA) positions, outlined in *pink*], leaving behind small intracellular stretches at both ends, and most part of the protein is extracellular (enclosed in a *dashed box*). Seven atypical calcium-binding motifs (*yellow boxes*) were present at regular intervals in the extracellular part of the *Pb*TIP containing the conserved D-x-N/D-x-D-xxx-D sequence. The VCBS (*Vibrio*, *Colwellia*, *Bradyrhizobium*, and *Shewanella*) domain was also present and encompassed the third and fourth calcium binding motifs (highlighted in *blue*). “*” indicates perfect alignment. Mutiple stars **, ***, ****, ***** are repetition of same marking and has same meaning as the single star. “:” indicates a site belonging to a group exhibiting strong similarity. “.” indicates a site belonging to a group exhibiting weak similarity. A putative epidermal growth factor (EGF)-like domains (shaded black) containing the RGD (arginine–glycine–aspartic acid) motif was also present between the fifth and sixth calcium-binding motifs. **(C)** The *Pb*TIP putative EGF-like domain aligns well with the mouse EGF and MSP1 of *P. berghei*, and these domains are well known to play an adhesion role in many *Plasmodium* proteins. The RGD motif further strengthens its role in adhesion.

BLAST searches of human TIP (Q8TB96) across species showed homology with a hypothetical *Plasmodium falciparum* protein, Q8I3H7, and both proteins shared 29.6% homology along the protein length (28). SMART domain analysis (Simple Modular Architecture Research Tool; http://smart.embl-heidelberg.de/) (29) revealed a conserved domain organization in human TIP and Q8I3H7, both containing transmembrane (TM) helices at similar locations. It also revealed the presence of the VCBS domain (pfam13517) (**Figure 1A**), which is found in several copies in long proteins in many species of *Vibrio*, *Colwellia*, *Bradyrhizobium*, and *Shewanella*, hence named VCBS. The suggested role of the VCBS domain is in cellular adhesion (30, 31). Through homology searches, we also found that *P. falciparum* Q8I3H7 is orthologous to an uncharacterized *P. berghei* protein, sharing 71.9% homology (query cover = 92%, *e*-value = 0.0). Human TIP also shares 27.88% homology with this *P. berghei* protein; hence, we called it *Pb*TIP (**Figure 1B**). *Pb*TIP also shares 28.33% homology with mouse TIP (Q99KW9.2). *P. falciparum* Q8I3H7 is not only an ortholog of *Pb*TIP; rather, other orthologs of this protein are present across all *Plasmodium* species. No paralogs of this protein could be found in respective *Plasmodium* species (32).

The adhesion property of human TIP and *Pb*TIP is envisaged by the presence of seven FG-GAP repeats, which is characteristic of integrin-α. The FG-GAP repeats also contained a conserved calcium-binding motif (D-x-N/D-x-D-xxx-D), and these repeats were shown to have importance in ligand binding (33, 34) (**Figure 1B**). A putative epidermal growth factor (EGF)-like domain is also present in *Pb*TIP, which aligned well with mouse EGF and the EGF-like domain of the *P. berghei* protein, MSP1 (merozoite surface protein 1) (**Figure 1C**). *Plasmodium* proteins containing an EGF-like domain have already been shown to mediate adhesion through this domain. The RGD motif (arginine–glycine–aspartic acid, a tripeptide motif that mediates adhesion in extracellular matrix proteins such as fibronectin, vitronectin, and laminin) in the EGF-like domain also strongly correlates with its role in adhesion (35). The existence of a VCBS domain in *Pb*TIP further strengthens its role in adhesion as the VCBS domain is implicated in cellular surface binding, as reported in various bacteria (30) (**Figure 1B** and **Supplementary Figure S1**). All the predicted domains of *Pb*TIP are summarized in **Figure 1A**.

### *Pb*TIP Localizes to the Surface of Infected Erythrocytes

**Figure 2:**
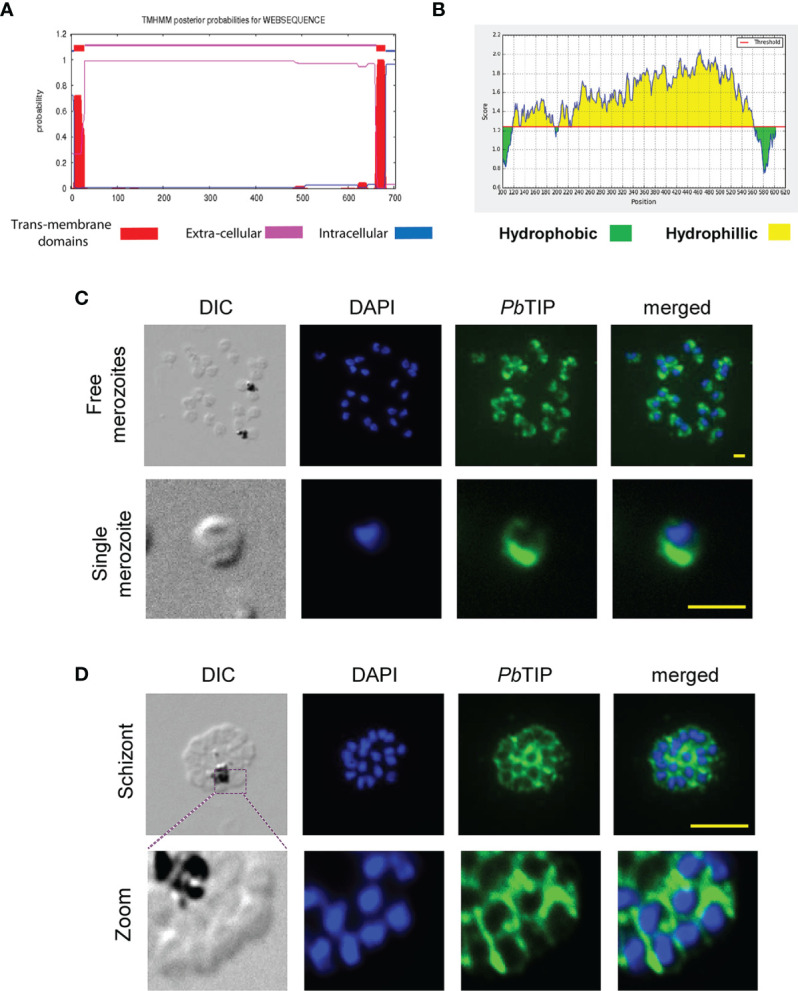
The *Plasmodium berghei* T-cell immunomodulatory protein (*Pb*TIP) is likely a membrane-anchored protein of merozoites. **(A)** The TMHMM server 2.0 predicts the presence of two transmembrane (TM) regions at both termini of this protein, while the intervening part between these TM helices is predicted as extracellular, suggesting that *Pb*TIP could be an extracellular membrane-anchored protein. **(B)** Parker’s hydrophilicity prediction showing two hydrophobic regions (*green*) at both termini corresponding to the TM helices, while the hydrophilic region (*yellow*) represents the extracellular part of the protein (window size = 200 amino acids, threshold = 1.243, center position = 100). **(C)** *Pb*TIP localization on the surface of *Plasmodium* merozoites as revealed by immunostaining against the target protein of formaldehyde-fixed free merozoites. The protein is abundant at one end of merozoites. *Scale bar*, 1 µm. **(D)** *Pb*TIP immunolocalization on the surface of mature schizont and around the merozoites packed within it. *Scale bar*, 10 µm.

TMHMM 2.0 (the transmembrane helix prediction tool; http://www.cbs.dtu.dk/services/TMHMM/) (36) analysis of the *P. berghei* TIP (PbANKA_124360.0) amino acid sequence revealed the presence of two TM helices, one at each terminus (**Figure 2A**). Moreover, Parker’s hydrophilicity tool also predicted two hydrophobic stretches in *Pb*TIP that correspond to two predicted TM helices at both termini (**Figure 2B**). The intervening part of the protein between two TM regions is likely extracellular and represents the ectodomain part of the protein (**Figure 1B**, dashed box).

Bacterially expressed recombinant *Pb*TIP was used for mouse and rat immunizations in order to raise immune sera against the protein. Immune serum was used to localize PbTIP in the parasite by performing immunofluorescence assays (IFAs). Our results suggest that *Pb*TIP was expressed on the surface of merozoites. Immunostaining images demonstrated that the protein localized to the merozoite surface, similar to MSP1, which is a known merozoite surface protein. Although its surface expression was not uniform like MSP1, it was abundant at the one end of merozoites (**Figure 2C** and **Supplementary Figure S2**). *Pb*TIP was also expressed in packed merozoites inside a schizont as a surface protein, clearly marking boundaries (**Figure 2D**).

**Figure 3:**
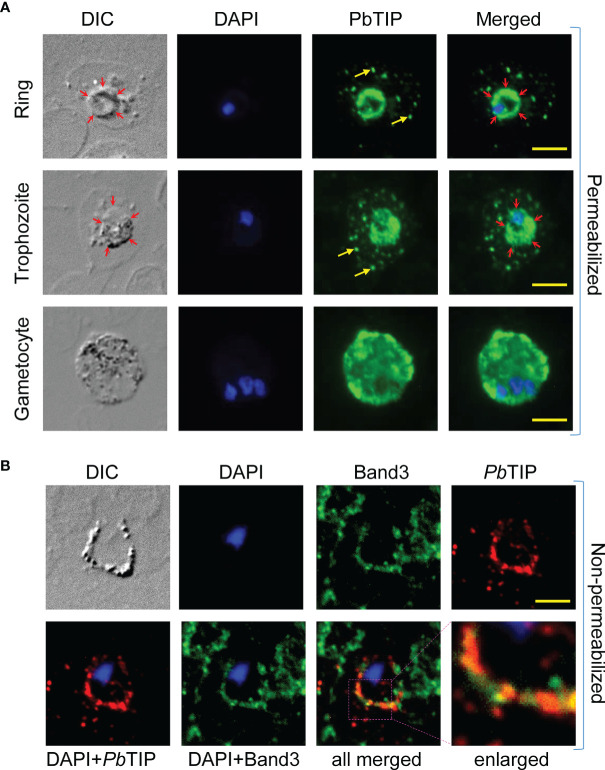
*Plasmodium berghei* T-cell immunomodulatory protein (*Pb*TIP) was exported across the parasitophorous vascular membrane (PVM) and into the infected surface of host erythrocytes. **(A)** Immunostaining of permeabilized *P. berghei* blood stages with antibodies (polyclonal sera) against the target protein revealed *Pb*TIP expression in its various forms. Intra-erythrocytic *Pb*TIP staining revealed its presence in the cytoplasm of host cell. *Yellow arrows* represent the *Pb*TIP in the cytosol of host cell in a punctate pattern. To demonstrate the export here, PVM is marked with *red arrows* (DIC panels) and the presence of *Pb*TIP beyond the PVM marked as the exported protein. Moreover, EXP1 (a known parasite PVM protein) and *Pb*TIP co-staining clearly demonstrated its export across the PVM in infected red blood cells (RBCs) (**Supplementary Figure S3**). *Pb*TIP is also localized to the PVM in the ring and trophozoites stages. *Pb*TIP expression in mature gametocytes was much higher compared to that in the ring and trophozoite stages. Permeabilization of blood smears was performed with 0.2% saponin, as described in *Materials and Methods*. **(B)** The export of *Pb*TIP into the surface of infected RBCs was demonstrated by performing immunostaining on non-permeabilized cells. *Pb*TIP (red fluorescence) can be seen clearly displayed on the infected surface of RBCs as a ring that co-localizes with band 3 (green fluorescence), which is one of the most abundant membrane proteins of erythrocytes. Band 3 antibodies were used to mark the surface of erythrocytes. This representative image was taken from more than 50 similar images. DAPI staining was used to locate the nucleus of the parasite. *Scale bar*, 5 µm.

Immunostaining of parasitized blood smear upon its permeabilization revealed that *Pb*TIP was localized to the parasitophorous vacuolar (PV) membrane and within the infected erythrocyte cytosol. *Pb*TIP was exported from the parasite into the host cell cytoplasm and surface, as exhibited by the immunostaining assay (**Figures 3A, B**). Intra-erythrocytic staining revealed the punctate pattern of *Pb*TIP in the ring and trophozoite stages of *P. berghei*. *Pb*TIP was also expressed in the gametocyte stage, and its expression was much higher in gametocytes compared to that in the asexual blood stages of *P. berghei* (**Figure 3A**, bottom panel).

When immunostaining was performed on a non-permeabilized infected blood smear, it showed *Pb*TIP expression on the surface of infected RBCs. The co-localization of band 3 (a RBC membrane marker) and *Pb*TIP indicated that this protein decorated the surface of infected host cells (**Figure 3B**). To be localized on the surface of erythrocytes, *Pb*TIP would have been exported across parasite confinement within the cell. The protein traversed the PV membrane and the host cytoplasm in order to reach the surface (**Supplementary Figure S3**). However, the amino acid sequence of *Pb*TIP did not show any identifiable *Plasmodium* export element (PEXEL) motif to facilitate its export, suggesting that it may be a PEXEL-negative exported protein (PNEP) (37).

### *Pb*TIP Is Processed and Shed From the RBC Surface

**Figure 4:**
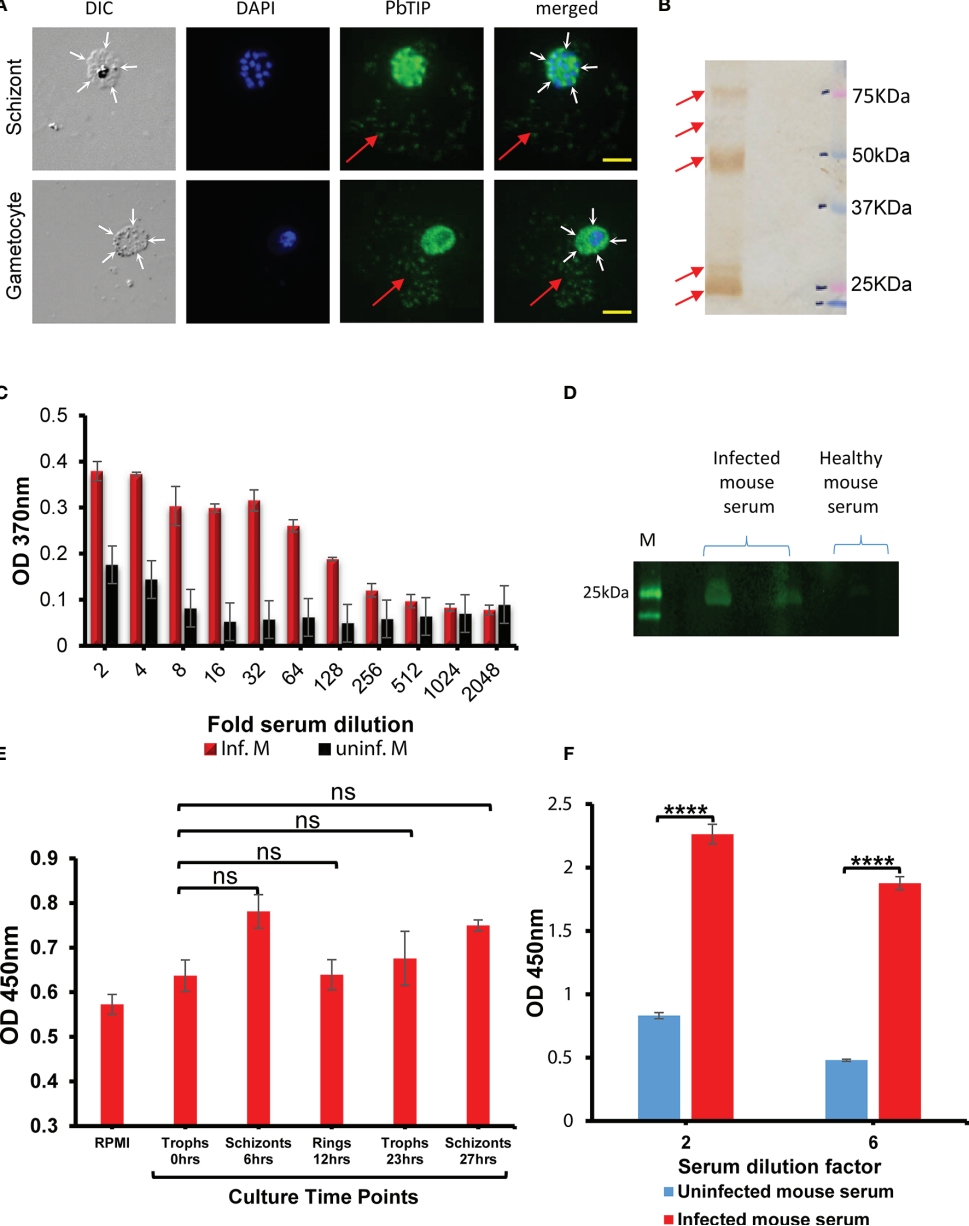
Parasitized erythrocyte surface-exported *Plasmodium berghei* T-cell immunomodulatory protein (*Pb*TIP) cleavage likely caused the shedding of this protein into host circulation. **(A)** Immunostaining of various forms of blood-stage parasites showed *Pb*TIP staining in their immediate surroundings. Immunostaining of the schizont and gametocyte stages showed shed *Pb*TIP in their surroundings. *Red arrow* represents the shed *Pb*TIP in the surroundings of these parasitic forms. The boundaries of infected host cells are marked with *white arrows*. These representative images were taken from more than 30 similar microscopy images. *Scale bar*, 10 µm. **(B)** Probing with anti-*Pb*TIP antibodies in the blood-stage parasite lysate revealed the fragmentation of *Pb*TIP into multiple smaller fragments. This fragmentation, by an uncharacterized process, is likely responsible for the shedding of the protein from the surface of erythrocytes. Full-length *Pb*TIP was observed at ~75 kDa, and its two major fragments were observed at ~50 and ~25 kDa. **(C)** Shed *Pb*TIP was detected in the infected host blood sera using sandwich ELISA, with healthy mouse sera as a negative control (*black bars*). *Pb*TIP could be detected in the host circulation up to 128-fold dilution of the blood sera (*red bars*). *Error bars* represent the standard deviation of the mean absorption at 370 nm optical density (OD). The data presented are from three replicates performed. **(D)** Fragments of shed *Pb*TIP (~25–30 kDa) detected by Western blotting of infected mouse serum, with healthy mouse serum as a negative control. **(E)** Detection of the released *Pb*TIP in the *P. berghei* culture medium harvested at various time points. Parasite culture was set from infected mouse blood containing mostly trophozoite stages, and the spent culture medium was harvested at various time points as described in the figure and sandwich ELISA performed as described in the *Materials and Methods*. In *in vitro* conditions, *Pb*TIP shedding was not optimum. Only a non-significant (*ns*) increase in the shed *Pb*TIP levels post-schizont bursting (at 6 and 27 h) was observed. Fresh culture medium, RPMI, was used as the negative control in the ELISA. Schizont bursting was ascertained by Giemsa staining of a cultured schizont smear. **(F)** For comparison purposes, we detected shed *Pb*TIP in infected mouse serum again using sandwich ELISA and observed that shedding was significantly higher in *in vivo* conditions (*p* = 0.00014 and 4.39E−05, respectively). The *x*-axis represents the dilution of serum used in the ELISA, i.e., at 1:2 and 1:6 ratios. Uninfected mouse serum was used as the negative control in ELISA. The mice used for both studies had identical parasitemia. “****” denotes the P value less than 0.00005 to 0.00001 and ns means not significant.

Immunostaining of parasitized red blood cells demonstrated the occurrence of *Pb*TIP in the cell surroundings, indicating that it is probably shed from the infected RBC surface. A mature schizont and gametocyte shedding of this protein are shown in **Figure 4A**. MSP1 (a merozoite surface protein) did not exhibit protein shedding, indicating that only some surface proteins were shed, which included *Pb*TIP (**Supplementary Figure S4**). However, the mechanism of this shedding is unknown, and it is possibly executed by the cleavage of the membrane-anchored *Pb*TIP by proteases similar to rhomboid protease (38, 39). The shedding of *Pb*TIP into host circulation is likely executed by its fragmentation, as multiple fragments of protein were detected in the parasite blood-stage lysate when subjected to electrophoresis and Western blotting. Uncleaved *Pb*TIP was detected at ~77 kDa, while multiple fragments of it were detected at various sizes, viz. ~70, ~50, and ~25 kDa (**Figure 4B**). Recombinant *Pb*TIP also exhibited cleavage and resulted in protein fragments of similar sizes in *Escherichia coli* (**Supplementary Figure S5A**), and *Pb*TIP cleavage was not due to a proteolytic cleavage during the purification process. However, the mechanism of *Pb*TIP cleavage and the protease(s) involved are still unknown. Protein sequencing of one of the major *E. coli*-expressed *Pb*TIP fragments (~55 kDa) pointed at the presence of one of the cleavage sites near its N-terminus (**Supplementary Figure S6** and **Table S1**).

Shed *Pb*TIP was also detected in the serum collected from malaria-infected mouse with indirect sandwich ELISA using antibodies against this protein. We detected *Pb*TIP even up to a 128-fold dilution of the infected mouse serum (**Figure 4C**). Serum from uninfected mice was taken as a negative control in the ELISA. Moreover, the infected host sera, when subjected to electrophoresis followed by immunoblotting with antibodies against *Pb*TIP, showed ~25- to 30-kDa sized fragments of *Pb*TIP, while no band was observed in healthy mouse serum (**Figure 4D**). These bands likely originate from the cleaved fragments of the surface-anchored extracellular domain of the *Pb*TIP. To address the critical aspect of *Pb*TIP shedding, which is the time of its release, we interrogated by culturing *P. berghei in vitro* and collecting the spent medium at various time points in order to detect shed *Pb*TIP in it using ELISA. Our results indicated that, in culture conditions, the *Pb*TIP level did not significantly change over the entire time course (one complete cycle of growth), suggesting that *Pb*TIP shedding is not optimal in the *in vitro* conditions (**Figure 4E**). A non-significant enhancement in shed *Pb*TIP in the culture medium was observed during the schizont stage (6 and 27 h post-culture). However, the *in vitro* results significantly differed from our *in vivo* data, where we detected a significant amount of shed *Pb*TIP in the serum collected from infected mice (**Figure 4F**), indicating that, somehow, shedding has a comparatively high feasibility *in vivo* than in *in vitro* conditions.

In brief, our results indicated that *Pb*TIP shedding is not optimal in culture conditions, while a significant amount of *Pb*TIP could be detected in *in vivo* conditions. Our immunostaining images also indicated that schizont rupture events did not contribute significantly to the release of *Pb*TIP into the surroundings (**Supplementary Figure S7**).

### *Pb*TIP Binding on Macrophages Downregulate Th1 Cytokines While Inducing Th2 Responsive Genes *In Vitro*

**Figure 5:**
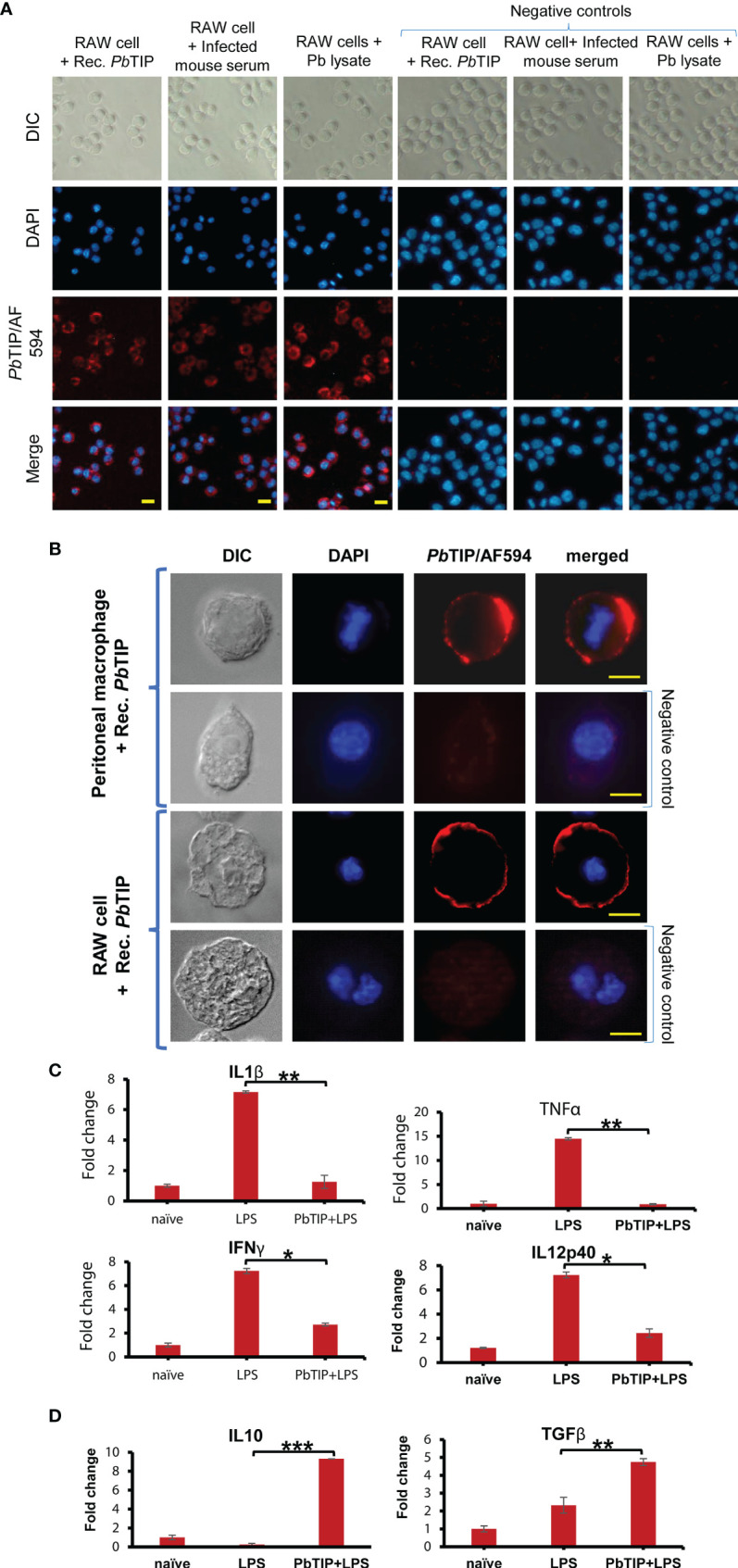
Shed *Plasmodium berghei* T-cell immunomodulatory protein (*Pb*TIP) in host circulation exhibited binding on macrophages and reduced their inflammatory responses while upregulating the Th2-type/anti-inflammatory responses *in vitro*. **(A)** Brief incubation of recombinant *Pb*TIP (10 µg/ml) with RAW 264.7 cells followed by immunostaining against the protein revealed its binding on the cell surface (*top left panels*). The shed *Pb*TIP in malaria-infected mouse serum also displayed binding on RAW 264.7 cells *in vitro* (*middle left panel*). No binding on RAW 264.7 cells was observed with healthy mouse serum (*right control panel*). Parasite blood-stage lysate (obtained from parasite free–thaw lysis) containing various fragments of *Pb*TIP also exhibited binding on RAW 264.7 cells (*bottom left panel*). Images were taken at ×200 magnification on a Zeiss Axio imager M2 microscope. DAPI was used to stain the nuclei of cells. *Scale bar*, 10 µm. **(B)** Recombinant *Pb*TIP binding upon incubation with murine peritoneal macrophages and RAW 264.7 cells. Images were taken at a higher magnification to unveil surface binding of *Pb*TIP. *Scale bar*, 10 µm (*top panel*) and 5 µm. Glutathione S-transferase (GST) protein was used as a negative control for binding. **(C)** *Pb*TIP surface binding reduced the ability of macrophages to mount inflammatory responses upon lipopolysaccharide (LPS) stimulation: (*i*) IL-1β was reduced by sixfold; (*ii*) TNF-α was reduced by 15-fold; (*iii*) IFN-γ was reduced by 2.6-fold; (*iv*) IL-12p40 was reduced by threefold. **(D)** The levels of anti-inflammatory cytokines such as TGF-β and IL-10, however, increased by five and ninefold, respectively, with longer exposure to *Pb*TIP (20 h). The experiment was repeated more than three times, and *C*_t_ values obtained from quantitative PCR (qPCR) were used to calculate the relative fold change using the 2^−ΔΔ^ *^C^* ^t^ method. Statistical significance values (*p*-values) of the transcript fold change between the LPS-stimulated and LPS+*Pb*TIP RAW 264.7 cells were given for various cytokine genes: for IL-1β, TNF-α, IFN-γ, and IL12p40, 0.0032, 0.008, 0.01, and 0.04, respectively. For the anti-inflammatory cytokines, such as IL-10 and TGF-β, the *p*-values were 0.0002 and 0.004, respectively. Student’s *t*-test was performed to calculate the statistical significance of the data. *, ** and *** signifies p values in the range of 0.05 to 0.01, 0.005 to 0.001 and 0.0005 to 0.0001, respectively.

The adhesion properties of *Pb*TIP in host circulation were evident *in vitro* by incubating recombinant *Pb*TIP (10 µg/ml) with murine origin macrophages, RAW 264.7 cells (**Figure 5A**, top panel). This binding is in concordance with various adhesion domains in *Pb*TIP (FG-GAP domains, RGD motif, VCBS domain, and EGF-like domain), which may affect binding on host cells (30, 35). *Pb*TIP binding on the surface of macrophages was further confirmed by incubating the sera of infected mice containing parasite-shed *Pb*TIP with RAW 264.7 cells (**Figure 5A**, middle panel). Parasite blood-stage lysate (obtained by freeze–thaw lysis) containing various fragments of *Pb*TIP also displayed binding on macrophages (**Figure 5A**, bottom panel). The binding was further confirmed using mouse peritoneal macrophages that demonstrated the surface binding of *Pb*TIP on macrophages (**Figure 5B**). Pre-immune serum of mice was used in negative controls while performing IFAs.

To study the immunomodulatory effect of *Pb*TIP upon its binding to host macrophages, we stimulated RAW 264.7 cells with *E. coli* lipopolysaccharide (LPS) in the presence and absence of recombinant *Pb*TIP. LPS is a well-known immune stimulus recognized through TLR4 present on the surface of macrophages leading to the activation of multiple signalling components, such as NF-κB and IRF3, and the subsequent production of Th1-type pro-inflammatory cytokines. In this section, the LPS-elicited immune responses of RAW 264.7 cells are described in the presence and absence of recombinant *Pb*TIP. We stimulated RAW 264.7 cells with *E. coli* LPS (100 ng/ml) alone or in combination with recombinant *Pb*TIP, and buffer (50 mM Tris, 150 mM NaCl, and 10% glycerol) alone was used as a negative control. LPS stimulation of RAW 264.7 cells for 9 h resulted in increased messenger RNA (mRNA) expression levels of the pro-inflammatory cytokines, which included interleukin 1β (IL-1β), IL-12, interferon gamma (IFN-γ), and tumor necrosis factor alpha (TNF-α). However, the levels of pro-inflammatory cytokines were reduced significantly when RAW 264.7 cells were treated with a combination of LPS and *Pb*TIP (10 µg/ml). The expression levels of cytokines were validated with quantitative PCR (qPCR) (performed in triplicate) at the transcript level, and the *C*_t_ values obtained were analyzed to calculate the relative fold change using the 2^−ΔΔ^ *^C^* ^t^ method. The transcript levels of IL-1β, TNF-α, IFN-γ, and IL-12p40 were reduced by 6-, 15-, 2.6-, and 3-fold, respectively (**Figure 5C**).

Furthermore, the stimulation of RAW 264.7 cells in a similar fashion for a longer duration, i.e., 20 h (40), resulted in the upregulation of Th2-type/anti-inflammatory cytokine transcripts while suppressing the inflammatory cytokine transcripts. The mRNA expression levels of TGF-β and IL-10 were upregulated by five and ninefold, respectively (**Figure 5D**). The cytokine transcript levels were validated by qPCR and analyzed in a similar manner (as in **Figure 5C**) to calculate the relative fold change. Details of the primers and their references are provided in **Supplementary Table S2**.

Furthermore, cytokines were also estimated in the cell culture medium (spent medium) of the LPS-elicited RAW 264.7 cells in the presence and absence of recombinant *Pb*TIP at respective time points through ELISA. Our results indicated that the expressions of the pro-inflammatory cytokines were suppressed significantly while those of the anti-inflammatory cytokines were found significantly upregulated in the study, conveying the same notion of immunosuppression and inducing Th2 responses in macrophages upon exposure to *Pb*TIP (**Supplementary Figure S8**).

### *Pb*TIP Accelerates Parasite Growth While Inducing Key Immunosuppressive Genes *In Vivo*

**Figure 6:**
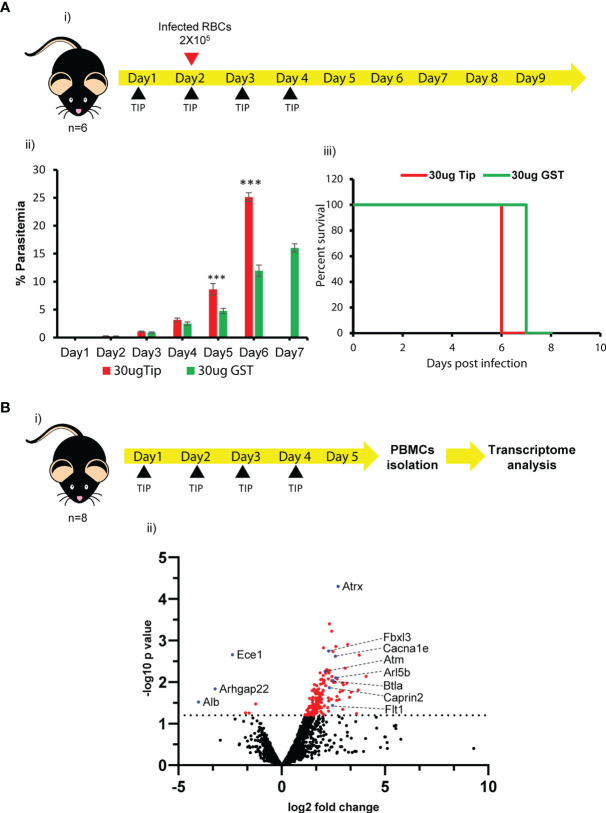
Recombinant *Plasmodium berghei* T-cell immunomodulatory protein (*Pb*TIP) treatment caused accelerated parasite growth in mice and systemic effect on host immune cells. **(A)** (*i*) Experimental workflow showing treatment of mice with recombinant *Pb*TIP to study its effect on parasite growth. The recombinant *P. berghei* protein was intraperitoneally injected (2 mg/kg) into C57BL/6J mice (*n* = 8) for four consecutive days. Infection was initiated on day 2 and daily parasitemia was followed post-infection. (*ii*) Injection of recombinant *Pb*TIP in malaria-infected mice accelerated parasite growth, as estimated by monitoring daily parasitemia. On day 6 post-infection, parasitemia was approximately twofold higher in *Pb*TIP-treated mice. Glutathione S-transferase (GST) protein-injected mice were taken as the control since recombinant TIP has a GST fusion partner. The experiment was repeated more than three times. Student’s *t*-test was performed to evaluate the significance of the data (the *p*-values on days 3, 4, 5, and 6 were 0.038, 0.01, 0.0003, and 2.91E−08, respectively, suggesting significant differences in parasitemia between the two groups). (*iii*) Recombinant *Pb*TIP-treated mice also showed early death compared to control-treated mice. However, it was not found to be statistically significant using the log-rank test (*p* > 0.05). **(B)** (*i*) Systemic effect of *Pb*TIP on host responses was studied by treating C57BL/6J mice with recombinant *Pb*TIP (2 mg/kg) for four consecutive days, followed by peripheral blood mononuclear cell (PBMC) isolation and transcriptome analysis. (*ii*) Volcano plot displaying the differentially expressed genes revealed in the transcriptome analysis, and the genes that may have direct relevance in our study are highlighted as *blue dots*. Various upregulated genes upon *Pb*TIP exposure are reported to be negative regulators of immune responses (*Arl5B*, *BTLA*, and *Fbxl3*), mediators of apoptosis (*ATM*, *Atrx*, and *Caprin2*), and induced in cancerous conditions (*Cacna1e* and *Flt1*). Of the downregulated genes, *Arhgap22* may affect the migration of macrophages upon *Pb*TIP exposure. GST protein-treated mouse PBMCs were used as the control in the transcriptome analysis. *Black dots* represent genes that were not significantly changed in the study (−log10 *p* < 1.2), while *red dots* represent the significantly upregulated/downregulated genes (−log10 *p* > 1.2). The *dashed line* denotes the cutoff mark for the significance level (−log10 *p* = 1.2). We used unpaired *t*-tests assuming unequal variances to calculate the significance values of the genes in the study. *** means p value < 0.0004.

The *in vivo* effect of recombinant *Pb*TIP on parasite growth was established by injecting this protein (2 mg/kg, day 1 onwards). The experimental design, number of doses, and the amount injected were all similar to those published by Fiscella et al. (27). Mice (*n* = 8 per group) were infected with 2 × 10^5^ *P. berghei*-infected RBCs given intravenously on day 2. After four consecutive daily doses of recombinant *Pb*TIP, we observed accelerated parasite growth in treated mice when compared with the control group (**Figure 6A** (ii)). Parasitemia was determined by staining thin blood smears with Giemsa stain and counting the parasites per 100 RBCs using a light microscope. At least 2,000 RBCs were counted for each slide. On day 5, the parasitemia in the treated group was almost twofold higher than that in the control group. Moreover, recombinant *Pb*TIP-injected mice died 1 day earlier, as shown in **Figure 6A** (iii).

The immunosuppressive effects of *Pb*TIP, as evident *in vitro* on host macrophages (**Figures 5C, D**), was further explored *in vivo*. The systemic effect of *Pb*TIP on various host responses was studied by treating healthy C57BL/6 mice with recombinant *Pb*TIP (2 mg/kg) for four consecutive days, followed by peripheral blood mononuclear cell (PBMC) isolation and transcriptome analysis. Glutathione S-transferase (GST) protein-injected mice were taken as control. Next-generation sequencing (NGS) data analysis revealed 51 upregulated and 3 downregulated genes (**Supplementary Table S3**).

Many of the differentially expressed genes have direct relevance to this study, and some of them are described here (**Figure 6B (ii)** and **Supplementary Figure S9**). Upon *Pb*TIP exposure, Arl5b, which is a negative regulator of MDA5-dependent immune response, was upregulated, and its overexpression repressed the MDA5-induced activation of the interferon-β promoter (41). The B- and T-lymphocyte attenuator (BTLA) was another upregulated gene upon *Pb*TIP exposure, which behaved like PD1 and CTLA-4. BTLA displayed T-cell inhibition and was also a negative regulator of B-cell proliferation (42). Upregulation of ATM (serine–threonine kinase) in immune cells may cause apoptosis of monocytes. *Atrx* and *Caprin2* were also upregulated and reported to be linked with apoptotic pathways (43, 44). Genes such as *Cacna1e* and *FLT1* were also significantly upregulated under cancerous conditions, and evidence suggests that these genes could be crucial factors for the macrophage M2 phenotype in the tumor microenvironment (45, 46). Such macrophages display skewed immune responses toward M2-type/anti-inflammatory response. Conversely, Ece1, involved in the proteolytic processing of endothelin 1 peptide and produced by the macrophages in response to microbial stimulation, was downregulated. Another downregulated gene, *Arhgap22* (a rho GTPase), has been reported essential in lamellipodia formation in macrophages, and its downregulation may affect the migration of macrophages upon *Pb*TIP exposure (47). A few of the differentially regulated genes selected based on transcriptome data were validated by qPCR (**Supplementary Figure S10**). The qPCR results concur with the transcriptome data. Primer details are provided in **Supplementary Table S2**.

## Discussion

The real success of parasites in their host is majorly attributed to their ability to subvert the anti-parasitic host immune responses (48). In higher vertebrates, the immune system has evolved to recognize a vast array of foreign invaders in order to facilitate their elimination (49). On the other hand, pathogens have developed various immunomodulation strategies to effectively manipulate host responses and thrive successfully in their host (12, 25). For example, mouse malaria is characterized by the production of pro-inflammatory cytokines in the early phase of the infection and a reduced cytokine response in the face of continuing infection. Dendritic cells has also become less responsive to TLR-mediated IL-12 and TNF-α production while enhancing their ability to produce the immunosuppressive cytokine IL-10 (14). During the later stages of malaria infection, host immunity becomes refractory not only to *Plasmodium* antigens but also to unrelated antigens (50). Immunomodulation by host molecular mimics has been reported during viral infections and is crucial during *Plasmodium* infection (51). *Plasmodium*-released host-like molecules, such as PMIF (*Plasmodium* macrophage migration inhibitory factor) and TCTP (translationally controlled tumor protein homolog of host histamine-releasing factor), have already been proven to manipulate the host immune responses for the benefit of the parasite (22, 52).

In the search of parasite molecules homologous to their host, we came across a T-cell immunomodulatory protein (TIP) that is conserved in *Plasmodium* species and shares homology with human TIP in its topology and domain structures (28). The presence of seven loosely conserved FG-GAP domains, including the conserved Ca^+^-binding motifs such as integrins of metazoans, suggests its role in adhesion. Reports suggested that these seven FG-GAP domains may fold into a seven-bladed beta-propeller structure that serves as its ligand-binding domain. *Pb*TIP is also conserved in metazoans from placozoa to vertebrates, including humans (53). All its orthologs across the species in *Homo sapiens* (ITFG1/TIP, 612AA), *Mus musculus* (ITFG2, 610AA), *Drosophila melanogaster* (CG7739, 596AA), and *Caenorhabditis elegans* (LNKIN1, 599AA) have similar domain organization and protein lengths. However, considering the relatively low homology between *Plasmodium* TIP and its natural host’s mosquito vector and human, the possibility of a recent horizontal gene transfer seems highly unlikely. It could be an old conserved protein that has undergone gradual changes, yet showing homology across the species. As described earlier, *Pb*TIP is conserved among all *Plasmodium* species; however, no paralogs have been reported, contrary to its host homolog integrins that are organized in multigene families with multiple paralogs (54).

Here, we report that *Pb*TIP is a surface protein of merozoites in *P. berghei* similar to MSP1; however, its expression was not uniform all over the surface of merozoites. It was localized abundantly at one end of the merozoites. The presence of two putative TM helices at each terminus further strengthens the *Pb*TIP membrane localization potential. *Pb*TIP was decorated on the surface of infected erythrocytes when merozoites developed into the ring and trophozoite stages. Our data indicated that *Pb*TIP was exported across the PV membrane, although it does not contain any recognizable PEXEL motif to facilitate export. Possibly, it is a PEXEL-negative exported protein, like many other *Plasmodium* proteins such as MAHRP1, SBP1, and REX2, where the N-terminal TM helix along with the few proximal amino acids are implicated in the export (37). The *Pb*TIP N-terminal TM helix along with a few amino acids may have facilitated its export in a PEXEL-negative manner. In the ring and trophozoite stages, *Pb*TIP decorated the parasite PV membrane within the infected RBCs, and it was also observed in the host cell cytosol. It could be possible that *Pb*TIP has become part of the PV membrane during its export to the host cell surface. Immunostaining of various asexual stages against *Pb*TIP further revealed that this protein was somehow shed from the surface of parasitized host cells, as we detected it in their surroundings. Protein shedding was seen significantly higher in the mature gametocyte stages. A few well-characterized *Plasmodium* proteins were shed by protease processing at the cellular surface, and these included AMA1, CSP1, and MSP1 (55, 56). CSP is known to undergo protease processing and shed during the gliding motility of sporozoites, leaving behind its trails (57). *Pb*TIP also sheds from the infected host cell surface as its multiple cleaved fragments were observed in the blood-stage parasite lysate with immunoblotting. Fragments of the same size were detected by immunoblotting in every independent experiment, suggesting that fragmentation did not result due to random protease processing. It is possibly cleaved by specific protease(s) that produces the same sized fragments every time.

There could be a variety of proteases involved in *Pb*TIP proteolytic processing and its subsequent shedding; however, the involvement of *Plasmodium* intramembrane rhomboid proteases is highly anticipated. Various intramembrane proteases have been characterized in Apicomplexa, including *Plasmodium* species that are implicated in the proteolytic cleavage of integral membrane proteins (58). The role of rhomboid protease in the shedding of TRAP during sporozoite motility and infectivity has been reported (38). Similarly, the shedding of AMA1 and MSP1 through the proteolytic activity of rhomboid proteases has been reported earlier. The existence of loosely conserved helix destabilizing residues in the N-terminal TM helix of *Pb*TIP also strengthens our prediction regarding the involvement of parasite rhomboid(s) in its cleavage and the subsequent shedding from the cellular surface (59). However, some of our findings unveiled that *Pb*TIP shedding is comparatively more feasible *in vivo* than in cultured conditions, suggesting the involvement of host origin factors in the shedding process. However, no direct evidence is available to support this; the mechanism of proteolysis and the proteases involved remain to be explored.

*Pb*TIP could also be detected in host circulation by immunoassays. In circulation, 25- to 30-kDa protein fragments were more abundant than were other fragments. Furthermore, the presence of TIP (from human malaria parasites) in host circulation could be exploited in the development of malaria rapid antigen testing kits. *Plasmodium* histidine-rich protein 2 (HRP2), lactate dehydrogenase (pLDH), and aldolase are extensively used for this purpose and pose few advantages over conventional microscopic testing of malaria besides sensitivity (60). Among all, HRP2 is predominantly used for rapid detection test (RDT) worldwide, but recent reports of HRP2 and HRP3 mutant *P. falciparum* parasites raised alarm over the search for alternative candidates for the purpose of RDT. The use of purified monoclonal antibodies against *Pf*TIP/*Pv*TIP will surely improve the ELISA sensitivity and bring the detection limit better than that of the present standards.

As described earlier, *Pb*TIP possesses integrin-α domains and various other adhesion domains (VCBS domain, EGF-like domain, and RGD motif). The presence of *Pb*TIP in host circulation directed us to look for the host cells on which it can possibly bind. We tested immune cells firstly as its homolog has already been proven to possess an immune modulation function. *Pb*TIP exhibited binding on macrophages; upon further analysis, we found that it was binding to macrophages of murine origin only. No binding was observed in cells of human origin, suggesting species specificity (data not shown). Furthermore, the *Pb*TIP receptor on macrophages was still elusive and out of the various adhesion domains, which one has affected the binding is still not clear. The receptor for the mammalian TIP is also not yet reported, therefore making it difficult to predict any strong candidate for *Pb*TIP binding.

Since we were more interested in the downstream effects of *Pb*TIP binding on macrophages to establish its possible role in immunomodulation, we tested the inflammatory responses of murine macrophages in the presence of recombinant *Pb*TIP. Exposure of macrophages to recombinant *Pb*TIP suppressed the inflammatory response while it upregulated the Th2-type/anti-inflammatory response. Inflammatory cytokines such as IL-1β, IL-6, IL-12p40, TNF-α, and IFN-γ were downregulated manifold. On the other hand, the anti-inflammatory cytokines such as IL-10 and TGF-β were upregulated manifold upon protein exposure to *Pb*TIP presence along with *E. coli* LPS. However, the fold change in cytokines at the transcript level (by qPCR) should not be compared with the corresponding ELISA data (protein level) as the qPCR data displayed the transcript profile of RAW 264.7 cells at the time of harvest while ELISA estimated the total accumulated protein in the spent media. Th*e* effect of parasite protein on macrophages leading to altered immune response may have direct relevance to the survival of parasites in the host. *Em*TIP, a homolog of human TIP in the tapeworm *Echinococcus multilocularis*, was also present in the excretory/secretory products of the parasite, and it has been shown to stimulate CD4^+^ T cells *in vitro* (61).

It is well established that enhanced levels of TGF-β and IL-10 lead to the increased number of Tregs. In malaria patients, higher levels of Tregs have been reported, which eventually developed tolerance in the host (62). *Pb*TIP may induce Tregs through IL-10 and TGF-β response by macrophages upon its binding, although it is too early to make such a statement. In the infection setting, macrophages and DCs are the primary cells that detect parasites mounting a pro-inflammatory cytokine response. Inflammation plays three major roles: phagocytosis, antigen presentation, and initiation of immune responses through the production of various cytokines and growth factors (6). A reduced inflammatory potential upon *Pb*TIP exposure may affect several aspects of macrophages, as explained, although we have not looked into their altered phagocytic activities or antigen presentation, if any. It appears that *Pb*TIP might have affected the NFκB and MAP kinase pathways that LPS activates through TLR-mediated binding to mount an inflammatory response. Reduced inflammatory responses from DCs and macrophages have been reported in murine malaria during the later stage of infection, as described earlier. Such responses from macrophages upon *Pb*TIP exposure seem to cause less Th1-type response while enhancing the ability of target cells to produce Th2-type cytokines. This may induce tolerance in the host in the long term, and the host becomes tolerant not only to parasite antigens but also to unrelated antigens. Recently, two groups [Guha et al., human field data (63), and Nahrendorf et al., mouse model data (64)] have shown that monocytes from malaria-exposed individuals differentiate into anti-inflammatory M2-type macrophages. Although their views differed on the locations where monocytes changed to the M2 phenotype, both groups agreed that these changes occurred through epigenetic modifications (a decrease in H3K4me3) at the inflammatory cytokine gene loci. The cytokine profile of malaria-infected RBC-exposed monocytes, described by Guha et al., largely matches with our data on *Pb*TIP protein-exposed macrophages. Therefore, it is likely that, among the many factors causing monocyte reprogramming by infected RBC, *Plasmodium*-released *Pb*TIP could be a leading cause.

The *in vivo* systemic effect of *Pb*TIP on various immune cells was studied in mice treated with recombinant *Pb*TIP, followed by PBMC isolation from the peripheral circulation and transcriptome analysis. The *in vitro* response of recombinant *Pb*TIP on macrophages should not be compared with its systemic *in vivo* effect. The interplay of various immune cells and their immune responses cannot be replicated *in vitro*; hence these studies cannot be directly correlated. Our analysis suggests that *Pb*TIP exposure upregulated the various immune inhibition genes or immune checkpoint pathway genes. Various apoptotic genes were found upregulated, which may have caused apoptosis of responsive immune cells. A few genes that skewed macrophages toward the M2 phenotype were also found differentially expressed upon *Pb*TIP exposure. Mice treated with recombinant *Pb*TIP have enhanced daily parasitemia, suggesting that this protein may affect parasite survival *in vivo*. Overall, this study suggests an immunosuppressive role of *Pb*TIP in host responses, as evident in the *in vitro* and *in vivo* studies. The function of mammalian TIP is still elusive for us to state that parasite *Pb*TIP anyhow mimics the host’s TIP function similar to the host MIF mimicked by the *Plasmodium*-released PMIF. To the best of our knowledge, genetic manipulation in mice has revealed the role of mouse TIP (ITFG2) in the development of B- and T-cell responses. Deficient mice showed retention of B cells in the spleen and a lower concentration of immunoglobulin G (IgG) in plasma. It was found essential for the development of a normal germinal center and for adequate humoral response (33). Hence, as of now, it is very early to make a statement that denotes *Pb*TIP as a functional mimic of host TIP.

In summary, our data suggest that a host-like protein in *P. berghei* was expressed on the surface of merozoites and exported to the surface of parasitized host cells in the blood-stage infection. By an uncharacterized mechanism, it was cleaved in multiple short fragments that are likely responsible for its shedding from the host cell surface. Shed protein was detected in infected host circulation, and it can be pursued in the development of rapid testing of malaria. The *Pb*TIP shed into host circulation also possessed adhesion properties, exhibited binding on the surface of macrophages, and modulated their inflammatory responses. We report macrophages with reduced inflammatory responses upon *Pb*TIP exposure and the upregulation of Th2 cytokines such as IL-10 and TGF-β. Our *in vivo* studies also conveyed the same effect of *Pb*TIP on various immune cells. The limitations of our results are that we need to replicate our finding with human malaria parasites and test the TIP of human malaria parasites in order to develop a RDT kit.

## Materials and Methods

### Animals and Parasite Strains

Six- to 8-week-old male/female C57BL/6J mice were used in all the animal experiments. All experimental procedures were carried out following Institutional Animal Ethical Committee (IAEC) guidelines (IAEC project approval no. NII/IAEC 535/19). To carry out experiments, the animals were first intraperitoneally injected with 150 μl of a ketamine–xylazine mix to induce short-term anesthesia. The *P. berghei* strains used in this study were obtained from The Malaria Research and Reference Reagent Resource Centre (MR4). Frozen stocks of Pb_ANKA (MRA-671, BEI Resources) or Pb_ANKA-GFPcon (MRA-867, BEI Resources) strains were injected into 6- to 8-week-old mice to infect them with malaria. *Anopheles stephensi* mosquitoes were reared in a humid chamber maintained at 27°C, 70% humidity, and fed on sucrose (20%)-soaked cotton pads. Young female mosquitoes were fed on *P. berghei*-infected C57BL/6J mice and afterwards maintained at 19–21°C, 70% humidity. On the 18th day after blood feeding, infected mosquitoes again fed on healthy mice to complete the cycle. Sporozoites were obtained by dissecting the salivary glands on the 20th day and then used for *in vivo* and *in vitro* infection experiments.

### Overexpression, Purification of *Pb*TIP, and Antibody Generation

A codon-optimized *Pb*TIP gene fragment (corresponding to 29–660 amino acids) omitting two terminal TM domains was cloned into the *E. coli* expression vector pGEX6p1, and ligation was facilitated using the *Bam*HI and *Sal*I restriction enzymes (NEB, Ipswich, MA, USA). Details of the primers used for cloning are given in **Supplementary Table S3**. The protein was overexpressed in BL21 cells (Stratagene, La Jolla, CA, USA) at 16°C for 15 h with 0.3 mM isopropyl β-d-1-thiogalactopyranoside (IPTG) at OD_600_ = 0.8. The recombinant protein was isolated from inclusion bodies by lysing the IPTG-induced bacterial pellet in lysis buffer (50 mM Tris–HCl, pH 8.0, 150 mM NaCl, 5 mM EDTA, 1 mM PMSF, and 10 µg/ml DNase), followed by sonication (amplitude = 30, pulse on = 5 s, pulse off = 10 s) and centrifugation. The supernatant was discarded and the pellet washed three times with wash buffer I (100 mM Tris–HCl, pH 8.0, 5 mM EDTA, 1 mM DTT, 2 M urea, and 2% Triton X-100); the pellet was finally resuspended in solubilization buffer II (50 mM Tris–HCl, pH 8.0, 150 mM NaCl, 1 mM DTT, and 8 M urea) at room temperature for 3–4 h. The clear supernatant was collected and passed through a 0.45-µm syringe filter, followed by sodium dodecyl sulfate polyacrylamide gel electrophoresis (SDS-PAGE) to check the purity of the protein. Urea was removed by stepwise dialysis, gradually decreasing the urea concentration in the dialysis buffer. No precipitate was observed during dialysis. No columns or purification matrix was used as the inclusion bodies itself gave highly pure protein. Purified protein treatment with Pierce™ High Capacity Endotoxin Removal Spin Columns (Thermo Scientific, Waltham, MA, USA) resulted in a protein preparation where the endotoxin level was below the recommended limit for animal use. Mice and rats were immunized with recombinant *Pb*TIP as per the standard immunization regimen given in **Supplementary Table S4**. A week after the final immunization, mouse sera were collected and the antibody titer was determined with direct ELISA (**Supplementary Figure S7**).

### Immunofluorescence Assays

Cells fixed on glass slides were permeabilized (if needed) with 0.2% saponin (Sigma, St. Louis, MO, USA) for 30 min, followed by blocking with 3% bovine serum albumin (BSA). The cells were incubated with primary antibodies (rat anti-*Pb*TIP or mouse anti-*Pb*TIP immune sera) at 500 dilutions overnight, followed by secondary antibodies (anti-rat AF594 or anti-mouse AF488; Abcam, Cambridge, UK) used at 2,000 dilution. Three phosphate-buffered saline (PBS) washes for 5 min each were given after incubations with the primary and secondary antibodies. Slides were mounted in anti-fade (50% glycerol and 2% DABCO; Sigma) and the images taken with a Zeiss AxioImager M2 fluorescence microscope.

### Western Blotting

Parasite pellets (obtained upon saponin lysis of infected RBCs) were resuspended in 1× PBS and sonicated to separate the soluble and insoluble fractions. The resulting pellet was solubilized in 0.1% SDS containing PBS and centrifuged to obtain the lysate. For Western blot of mouse serum, the excess of albumin protein was removed with AlbuminOut (G-Biosciences, New Delhi, India), followed by SDS-PAGE. The protein from the gel was transferred into a nitrocellulose membrane (Bio-Rad, Hercules, CA, USA), followed by overnight incubation with a blocking buffer (Odyssey). The membrane was probed with a primary antibody (rat anti-*Pb*TIP immune sera, 1/1,000 dilution) for 3 h, followed by an IR-labeled secondary antibody (rabbit anti-rat IR800, 1/10,000 dilution; Invitrogen, Carlsbad, CA, USA) for 2 h. The blot was imaged using the Licor Odyssey imager. For the horseradish peroxidase (HRP)-labeled secondary antibodies, the membrane was incubated with 10 ml of 0.1% 3,3′-diaminobenzidine tetrahydrochloride (DAB; Sigma) containing 12 µl of 30% H_2_O_2_ until the band developed.

### Sandwich ELISA

A high-protein-binding 96-well ELISA plate (Corning, NY, USA) was coated with mouse anti-*Pb*TIP immune sera in coating buffer overnight at 4°C. Serial dilutions of the *Plasmodium*-infected mouse sera were incubated overnight for binding in cold conditions, followed by incubation with the rat anti-*Pb*TIP immune sera (primary antibodies, 1,000 dilution) for 2 h the next day. The rabbit anti-rat HRP secondary antibody (Novus Biologicals, Englewood, CO, USA) was used at 5,000 dilution for 1 h and developed by adding 100 µl/well of the TMB substrate (Sigma–Thermo Fisher, India), and the absorbance was measured at 370 nm in a Tecan M200 (Theale, Reading, UK) plate reader. In the direct ELISA for estimating the antibody titer, 0.2–0.5 µg/well of the recombinant *Pb*TIP was coated; the rest of the process remained the same.

For cytokine estimation using ELISA, the spent culture medium was collected at 9 and 20 h and 50 µl of the spent medium was used for ELISA following the manufacturer’s protocol (BioLegend cat. nos. 432604, 431604, 436707, and 431411; Becton Dickinson cat nos. 560478 and 555138).

### Parasite Culture for Schizonts

C57BL/6J mice infected with *P. berghei* parasite were humanely sacrificed at 8%–10% parasitemia (mostly trophozoites); the infected blood was collected in heparinized vials. Blood was centrifuged to remove plasma, resuspended in complete RPMI1640 [20% fetal bovine serum (FBS) and 50 mg/L hypoxanthine) media, keeping the hematocrit at 4%–6%, and incubated at 37°C. The culture flasks were flushed with the excess of mixed gas (90% N_2_, 5% CO_2_, and 5% O_2_) and capped tightly. At regular intervals, the spent culture medium was collected, centrifuged, and kept for ELISA. Schizont formation and their bursting were monitored with Giemsa staining of thin smears of blood culture collected at regular intervals.

### Macrophage Binding Assay

Thioglycolate elicited mouse peritoneal macrophages were isolated in ice-cold Dulbecco’s PBS and allowed to adhere onto sterile coverslips on a 24-well cell culture plate containing complete RPMI medium (Lonza, Basel, Switzerland). One hour later, the cells were washed with PBS to remove non-adherent cells. Similarly, RAW 264.7 cells were also seeded in a cell culture plate and 10 µg/ml of the recombinant *Pb*TIP was incubated with peritoneal macrophages and RAW 264.7 cells for 1 h. After incubation, the cells were washed with an excess of PBS and fixed with 4% paraformaldehyde (PFA), followed by IFAs to decipher the surface binding of *Pb*TIP.

### Macrophage LPS Stimulation *In Vitro*

RAW 264.7 cells were cultured in complete Dulbecco’s modified Eagle’s medium (DMEM; Lonza) containing 10% FBS (Gibco, Waltham, MA, USA) and seeded into a 24-well cell culture plate. The cells were stimulated with 100 ng/ml of *E. coli* LPS (Thermo Fisher, India) in the presence and absence of 10 µg/ml recombinant *Pb*TIP for recommended hours. Post-incubation, the RNA was isolated from each well with Trizol (Thermo Fisher, India) following the recommended procedure and 2 µg of the RNA was converted into complementary DNA (cDNA) with an iScript reverse transcription kit (BioRad). qPCR was performed with the gene-specific primers of the various cytokines and a high-efficiency qPCR mix (cat no. 18114R1074; GCC Biotech, West Bengal, India) to calculate the relative fold change (**Supplementary Table S3**). The primers used in the study are given in **Supplementary Table S3**.

### Transcriptome Analysis

A group of eight C57BL/6J mice were given four consecutive doses of 2 mg/kg of recombinant *Pb*TIP and their blood collected. An equivalent group of control-treated (GST protein-treated) mice were included in the experiment. PBMCs were isolated with Percoll-based density gradient centrifugation and total RNA was isolated from the purified PBMCs. Briefly, Trizol-preserved samples were stored in −80°C deep freezer until use and then thawed once at the time of RNA extraction. Chilled chloroform was added to one-fifth volume of the Trizol, mixed well, and centrifuged to collect the upper aqueous phase. RNA was precipitated with an equal volume of chilled isopropanol and retrieved by centrifugation at full speed. The RNA pellet was washed with chilled 70% ethanol, air dried, and dissolved in an appropriate volume of nuclease-free water. The RNA integrity number (RIN) was >7 for each sample. A TruSeq (Illumina, San Diego, CA, USA) stranded mRNA library was made using 5 μg total RNA and was sequenced with the Illumina NovaSeq 6000 platform. The paired-end read length was 150 bases. A total of 6 Gb data was collected per sample.

Initial RNA sequence quality assessment was carried out on raw reads using FastQC v0.11. Adapter contamination and low-quality reads (*Q* < 30) were trimmed out using the NGSQC Toolkit v2.3.3. If 70% of the reads had average good quality (*Q* ≥ 30), then it was kept and was considered as high-quality (HQ) data. In the next step, HQ reads were assembled using a *de novo* transcriptome assembly method. The total reads obtained were 37,546,250 and 37,546,250 for the control and 33,346,685 and 33,346,685 for the TIP-treated samples Differential gene expression analysis was carried out using Cuffdiff v2.2.1. The obtained blast hits were then used to annotate the transcripts at different levels, such as pathway and Gene Ontology (GO) annotation, using the Retrieve/ID mapping utility provided by the UniProt database.

### Giemsa Staining

Thin blood smears fixed on glass slides (methanol fixed) were stained with a 1:10 times diluted Giemsa staining solution (48900-1L-F; Sigma) in a staining buffer (8 mM KH_2_PO_4_, 6 mM Na_2_HPO_4_, pH 7.0). The Giemsa stain was kept on slides for 30 min, followed by washing the slides under running tap water to remove excess of the stain, and then the slides were air dried for some time. Blood smears were observed under ×100 magnification using oil immersion lens fitted in a Nikon 80i microscope.

### Statistical Analysis

Data are presented as the mean and SDs. Statistical analyses were performed using Student’s *t*-test. Statistically significant differences compared with the control are indicated by asterisks: *p < 0.05, **p < 0.01, ***p < 0.001. All the statistical analysis was done in Excel or GraphPad Prism. Survival curve was analyzed by the log-rank test to evaluate its statistical significance.

## Data Availability Statement

The datasets presented in this study can be found in online repositories. The names of the repository and accession number can be found below: https://www.ncbi.nlm.nih.gov/, GSE173309.

## Ethics Statement

The animal study was reviewed and approved by the Animal Ethics Committee of the National Institute of Immunology, New Delhi, India.

## Author Contributions

AS and RA conceived the project. IK and AS designed all the experiments and analyzed the data. IK performed the experiments. RA, SB, and BS generated the reagents for the study. AQ helped in transcriptome data collection and manuscript editing. All authors contributed to the article and approved the submitted version.

## Conflict of Interest

The authors declare that the research was conducted in the absence of any commercial or financial relationships that could be construed as a potential conflict of interest.

## Publisher’s Note

All claims expressed in this article are solely those of the authors and do not necessarily represent those of their affiliated organizations, or those of the publisher, the editors and the reviewers. Any product that may be evaluated in this article, or claim that may be made by its manufacturer, is not guaranteed or endorsed by the publisher.

## Supplementary Material

Supplementary Material Gene ontology (GO) analysis. Gene ontology (GO) analyses revealed that following cellular components were enriched; Plasma membrane (GO:0005886), Cytoplasm (GO:0005737), nucleus (GO:0005634) and integral components of membrane (GO:0016021). Similarly enriched molecular components include, identical protein binding (GO:0042802), ATP binding (GO:0005524), signalling receptor binding (GO:0005102) Calcium ion binding (GO:0005509) and Zinc ion binding (GO:0008270). The biological processes that were majorly affected included cell surface receptor signalling (GO:0007166), cAMP biosynthetic process (GO:0006171), negative regulation of angiogenesis (GO:0016525), intracellular signal transduction (GO:0035556) and cellular response to LPS(GO:0071222). Detailed analysis further revealed that up-regulated genes inhibit immune response; by negative regulation of i) antigen processing and presentation of endogenous peptide antigen *via* MHC class I *via* ER pathway, TAP-independent [GO:0002486], ii) CD4^+^ alpha-beta T cell proliferation [GO:2000562], iii) CD8^+^ alpha-beta T cell proliferation [GO:2000565], iv) IFN-gamma production [GO:0032689], v) IL-17 production [GO:0032700], vi) TNF production [GO:0032720], and by positive regulation of i) I-kappaB kinase/NF-kappaB signaling [GO:0043123], ii) IL-10 production [GO:0032733] iii) isotype switching [GO:0045830] iv) regulatory T cell differentiation [GO:0045591], v) protein ubiquitination [GO:0031398] vi) TGF beta receptor signaling pathway [GO:0007179] Upregulated genes are also involved in immune checkpoint blockade pathways as evident by enrichment of GO terms i) negative regulation of cell population proliferation [GO:0008285] ii) intracellular signal transduction [GO:0035556], iii) immune response-regulating cell surface receptor signalling pathway [GO:0002768] iv) extrathymic T cell selection [GO:0045062] and v) cell population proliferation [GO:0008283]

Supplementary Table 1 Peptides detected in MS/MS analysis of ~55kDa fragment of the recombinant *Pb*TIP. Peptide sequences revealed that 55kDa fragment is a cleavage product of full-length protein, hence it is the part of the protein.

Supplementary Table 2 Primers used in this study.

Supplementary Table 3 List of differentially expressed genes in mouse PBMC upon recombinant *Pb*TIP treatment. GST protein treated PBMCs was taken as control here.

Supplementary Table 4 Mouse or rat immunization regime to raise antibodies against *Pb*TIP.

Supplementary Figure 1 Smart domain analysis (http://smart.embl-heidelberg.de/) of *Pb*TIP amino acid sequence revealed the presence of VCBS domain positioned between 196 to 277 amino acid residues. The suggested role of VCBS domain is in the adhesion and it is present in multiple copies in the long proteins of many bacteria.

Supplementary Figure 2 Antibodies against the MSP1 stain the merozoites surface uniformly unlike *Pb*TIP. Anti-MSP1 primary antibody staining (secondary antibody was labelled with Alexa 594) showed that MSP1 was present uniformly all over the merozoites surface while *Pb*TIP expression was uneven on the merozoites surface. *Pb*TIP is localized more at one end of the merozoites rather than distribution all over the surface as MSP1. Scale bar 1µm.

Supplementary Figure 3
*Pb*TIP is exported across the PV membrane and localized to the surface of host cells. *Plasmodium Pb*TIP is exported out of PVM and become the part of erythrocytes membrane. Here, the PV membrane is marked in red by staining against the EXP1 protein (secondary antibody was labelled with Alexa 594) and *Pb*TIP expression (green, secondary antibody with Alexa 488) is shown beyond it. Scale bar 5µm.

Supplementary Figure 4 MSP1 is not shed from schizonts surface. MSP1 protein was used as a negative control to show shedding of *Pb*TIP from the *Plasmodium* schizonts and other stages of parasite. *Pb*TIP was shown to shed from various parasitic stages while MSP1 gives the typical staining on merozoites and schizonts. Scale bar 5µm.

Supplementary Figure 5 Expression and purification of recombinant *Pb*TIP in *E. coli*. **(A)** Inclusion bodies from IPTG induced bacterial pellet was solubilized in 8M urea buffer and protein recovered from inclusion bodies was refolded by step-wise removal of urea by dialysis. No precipitation was observed during dialysis and high purity (>95%) protein was recovered from inclusion bodies. Refolded protein was run on an acrylamide gel. Two lanes represent protein loading in duplicates. **(B)** Overexpressed recombinant *Pb*TIP was confirmed with western blotting using antibodies against the GST and detected the full-length *Pb*TIP along with GST at ~ 95kDa (right panel). A major fragment was observed at ~55kDa which is a cleaved fragment of the full-length *Pb*TIP and explained below. Two lanes represent the protein loading in duplicates.

Supplementary Figure 6 N-terminal sequence analysis of the ~55kDa fragment recovered along with full-length recombinant *Pb*TIP revealed one of the cleavage sites near its N-terminus. Inclusion bodies purified *Pb*TIP was subjected to electrophoresis and transferred onto the PVDF membrane. After Ponceau staining *Pb*TIP fragment ~55kDa (TF55) was cut from the membrane and processed for N-terminal amino acid sequencing by Edman`s degradation method. The first 10 amino acids of this *Pb*TIP fragment revealed a cleavage between128-129 amino acids position in the full-length protein.

Supplementary Figure 7 Schizonts burst do not contribute to the shed *Pb*TIP in the host circulation. Shedding of *Pb*TIP from a gametocyte (FITC signal) while signals were not detected from the merozoites upon schizonts burst. These two independent events were captured in the same field suggesting that schizonts burst does not contribute significantly to shed *Pb*TIP.

Supplementary Figure 8 Pro-inflammatory and anti-inflammatory cytokines profile of LPS elicited RAW 264.7 cells upon exposure of recombinant *Pb*TIP. RAW 264.7 cells were stimulated with LPS (100ng/ml) alone and LPS+recombinant *Pb*TIP (10µg/ml) keeping the untreated cells as naïve. The level of various cytokines was measured in the culture medium (spent medium) by ELISA following the manufacturers protocol (Biolegend and BD). ELISA results indicate that *Pb*TIP significantly suppressed the pro-inflammatory cytokines (IL-1β, TNF-α, IFN-γ and IL-12p40) expression in LPS elicited macrophages *in-vitro*, suggesting immunosuppressive effect of *Pb*TIP on macrophages. P-values for IL-1β, TNF-α, IFN-γ and IL-12p40 respectively are 0.0017, 0.0032, 0.0203, and 0.0026. Pro-inflammatory cytokines were measured upon 9hours of stimulation. Anti-inflammatory cytokines (IL-10 and TGF-*β*) were also measured by ELISA however upon longer incubation (20hours) and found to be significantly over-expressed in *Pb*TIP exposed LPS-elicited macrophages. These results demonstrate the *Pb*TIP potential to favour anti-inflammatory responses in macrophages while suppressing the pro-inflammatory responses. P-values for IL-10 and TGF-*β* respectively are 0.00504 and 0.0005. P-values were calculated by applying t-test.

Supplementary Figure 9 Volcano plot displaying differentially expressed genes of PBMCs upon *Pb*TIP treatment. The volcano plot displaying differentially expressed genes of mouse PBMCs upon treatment with *Pb*TIP as described in (i). Here the x-axis represents the log_2_ fold change while the y-axis denotes –log_10_ (q-value) or –log_10_ (false discovery rate). All significantly up-regulated/down-regulated genes are highlighted in red dots while genes that were validated by qPCR are in blue dots. Black dots denote non-significant genes in the study (adjusted –log_10_ q-value<0.05). Most of the genes in the study were found to be up-regulated hence giving a skewed appearance to the volcano plot.

Supplementary Figure 10 Transcriptome data validation by RT-qPCR. Primers were designed against the few genes that were significantly changed in the transcriptome data. Primers list is given in the **Table S3**. RT-qPCR was performed on cDNA from RNA samples that were given for transcriptome analysis. The genes transcripts that were found up-regulated in transcriptome analysis were also found up-regulated when tested by qPCR.

Supplementary Figure 11 Estimation of antibody titre of anti-*Pb*TIP immune sera. Antibody titre of anti-*Pb*TIP antibodies collected from recombinant *Pb*TIP immunized mice, was estimated by ELISA. ELISA plate was coated with purified recombinant *Pb*TIP and ELISA was performed as discussed earlier in the method section. OD370nm was plotted against different dilutions of immune sera. Antibodies against *Pb*TIP were able to recognize antigen up to ~0.25 million dilutions. Control immunized (sham-treated) mice were taken as a negative control in ELISA.
