# Supplementary material for: Plasmodium berghei-Released Factor, PbTIP, Modulates the Host Innate Immune Responses
Source: Front Immunol. 2021 Dec 7;12:699887. doi: 10.3389/fimmu.2021.699887 (PMC8721568; doi:10.3389/fimmu.2021.699887)
Supplement: Supplementary Table 1 — Peptides detected in MS/MS analysis of ~55kDa fragment of the recombinant PbTIP. Peptide sequences revealed that 55kDa fragment is a cleavage product of full-length protein, hence it is the part of the protein. [file DataSheet_2.pdf]

Table S1. Peptides detected in MS/MS analysis of ~55kDa fragment of the recombinant *Pb*TIP. Peptide sequences revealed that 55kDa fragment is a cleavage product of full-length protein, hence it is the part of the protein.

Peptide sequence	Position	AA	Frequency
LASNLcSESDFEFANK	379-394	16	137
NEQISNNDPNHTDIR	451-465	15	47
GLHFYGDASYPSYLSVGDVDDDGYLDLLVSLR	407-438	32	3
SQSAHSPLQLPYVLFGLGR	585-603	19	5
WQIQLSVNPSNK	648-659	12	4
IYKNEQISNNDPNHTDIR	448-465	18	13
ADLVFTVINK	273-283	12	27
LSPDYISLLDDK	395-406	12	14
STALNGIcVNDcYK	533-546	14	5
TLGGNAHGPTFK	555-566	12	23
IYFIPNIQTK	354-363	10	19
SNETDSLFLK	523-532	10	14
NEQISNNDPNHTDIRR	451-466	16	3
KYSSVGFVR	514-522	9	27
ITVIDANGTK	567-576	10	6
FIWINNGNDGFK	227-238	12	10
NDKGQNFVR	439-447	9	1
ADLVFTVINKK	273-283	12	4
SSScNYccTR	341-350	10	3
ADEDYmLPANSmQIIFADFNADGSIDmVVPTcVK	307-340	34	1

Table S2. Primers used in this study.

Primer name	Sequence 5`>>3`
Mouse TNFα_FP	TCAGCCGATTTGCTATCTCATA (66)
Mouse TNFα_RP	AGTACTTGGGCAGATTGACCTC
Mouse IL1β_FP	CCAAGCAACGACAAAATACC (67)
Mouse IL1β_RP	GTTGAAGACAAACCGTTTTTCC
Mouse IL6_FP	GAGGATACCACTCCCAACAGACC
Mouse IL6_RP	AAGTGCATCATCGTTGTTCATACA
Mouse IL10_FP	TGGCCCAGAAATCAAGGAGC (68)
Mouse IL10_RP	CAGCAGACTCAATACACACT
Mouse IL12p40_FP	GGAAGCACGGCAGCAGAATA
Mouse IL12p40_RP	AACTTGAGGGAGAAGTAGGAATGG
Mouse IFNγ_FP	GGCCATCAGCAACAACATAAGCGT
Mouse IFNγ_RP	TGGGTTGTTGACCTCAAACTTGGC
P. berghei 18s-rRNA_FP	AAGCATTAAATAAAGCGAATACATCCTTAC
P. berghei 18s-rRNA_FP	GGAGATTGGTTTTGACGTTTATGTG
IJK_30TF	GCCTCGAGATCTGCATTTATTGTCTTCA
IJK_31TF	CGGGATCCCTACCAGCTAATTCTATGCAA
P. berghei Hsp70_FP	TAGTATTATTTGTATCCCTG
P. berghei Hsp70_FP	CTTTTGCTACTTCTTTGTCATC
IJK_35Topt	ACTAGTCGACGGGGAAATTATACCGAGTT
IJK_36Topt	AAGGAAAAAGCGGCCGCTTTGTTAGATGGGTTCACTG
Btla_FP	CAGAACACCCACTAATAACAG
Btla_RP	GTGTAAAGCAGCCAAGTCCT
atrx_FP	GGTTCATCAAGGTCAAAGAGG
atrx_RP	CATCTTTGTCTTCATTCAGC
CD209c_FP	GTCTCTAATGTTGCCTACTC
CD209c_RP	CCAGTTCTGTTGGAACTTGGAG
CXCR2_FP	AGCTCTGGCATGCCCTCTATT
CXCR2_RP	AGGTTCAGCAGGTAGACATC
Fbxl3_FP	GAGGAAGAGATAGTGACCAG
Fbxl3_RP	GGAAGCATGAGCCCGGTCAAG
Flt1_FP	GATATGGCTCAGGGTCGAAG
Flt1_RP	CTTTTGTCCTCCTGGCTCAC
Clk4_FP	TCCAAACGAACTCACTGTCC
Clk4_RP	TCTCTCTCATTCAAGCATCT
carf_FP	CGTCATTCCTTAGCGGGCAAC
carf_RP	TCCTGTGATGTGAGTGAA

References for primers described in table-S2:

66. Sun S, Shi G, Sha H, Ji Y, Han X, Shu X, Ma H, Inoue T, Gao B, Kim H, et al. IRE1α is an endogenous substrate of endoplasmic reticulum-associated degradation. *Nat Cell Biol* (2015) **17**:1546. doi:10.1038/NCB3266

67. H S, S S, AB F, N E, Z X, L L, P L, F M, RD G, MS P, et al. The ER-associated degradation adaptor protein Sel1L regulates LPL secretion and lipid metabolism. *Cell Metab* (2014) **20**:458–470. doi:10.1016/J.CMET.2014.06.015

68. Hop HT, Arayan LT, Reyes AWB, Huy TXN, Min WG, Lee HJ, Rhee MH, Chang HH, Kim S. Heat-stress-modulated induction of NF-κB leads to brucellacidal pro-inflammatory defense against Brucella abortus infection in murine macrophages and in a mouse model. *BMC Microbiol 2018 181* (2018) **18**:1–12. doi:10.1186/S12866-018-1185-9

Table S3: List of differentially expressed genes in mouse PBMC upon recombinant *Pb*TIP treatment. GST protein treated PBMCs was taken as control here.

S.no.	Gene	FPKM_*Pb*TIP	FPKM_GST	log2(fold change)	p_value	q_value
1	2010007H06Rik	0.196593	1.74287	3.14818	0.0107	0.66754
2	4932438A13Rik	1.4417	6.88647	2.25599	0.0084	0.62567
3	A830080D01Rik	0.41858	2.07235	2.30769	0.0053	0.4984
4	Abl2	0.785355	3.16726	2.01182	0.00695	0.565095
5	Aff4	1.24025	6.61673	2.41549	0.0006	0.276187
6	AI504432	0.435676	3.3544	2.94473	0.0162	0.793258
7	Ankrd26	0.127665	1.07468	3.07348	0.00465	0.4984
8	Ankrd28	0.658779	2.75155	2.06238	0.0091	0.631441
9	Ap4e1	0.511537	2.52985	2.30614	0.0004	0.276187
10	Arl5b	0.304868	1.86266	2.61111	0.0079	0.601496
11	Atm	0.578951	2.47478	2.09579	0.00555	0.4984
12	Atrx	1.6747	11.0807	2.72607	5.00E-05	0.054883
13	AU041133	0.065745	0.672486	3.35454	0.0176	0.8099
14	BC051142	0.165562	1.31225	2.98661	0.0117	0.678909
15	Brwd3	0.153856	0.753897	2.29279	0.00605	0.52428
16	Btla	5.77979	32.059	2.47164	0.00925	0.631441
17	C130036L24Rik	0.177783	0.861281	2.27637	0.0285	0.91117
18	Cacna1e	0.678467	4.09984	2.59522	0.0024	0.426593
19	Caprin2	0.121011	0.598991	2.30739	0.0137	0.733563
20	Carf	0.067079	0.650006	3.27651	0.02325	0.877504
21	Ccdc171	0.248481	1.33074	2.42102	0.0093	0.631441
22	Ccnt2	1.852	7.59209	2.03541	0.00965	0.635549
23	Cd209c	0.068313	0.88926	3.70237	0.01555	0.775851
24	Cenpf	0.125055	0.882105	2.81839	0.01725	0.8099
25	Cep85l	0.128013	0.599218	2.22679	0.041	0.999566
26	Clk4	4.7344	19.2094	2.02056	0.0015	0.36974
27	Clock	0.296455	1.4258	2.26588	0.00575	0.508315
28	Col9a3	0.073942	1.24984	4.07921	0.0073	0.582761
29	Crebrf	1.30058	5.69512	2.13057	0.00615	0.529463
30	Cxcr2	0.864386	3.46588	2.00348	0.01575	0.779921
31	D130040H23Rik	0.082623	1.11351	3.75244	0.00225	0.426593
32	D3Ertd751e	0.258616	1.06712	2.04484	0.0105	0.66754
33	Ddx26b	1.41783	8.06823	2.50857	0.0103	0.661813
34	Dennd5b	0.529518	2.39523	2.17741	0.00505	0.4984

35	Dmxl1	0.559016	3.12133	2.4812	0.00185	0.393035
36	Dmxl2	0.118083	1.07545	3.18706	0.00125	0.36974
37	Dpy19l4	0.165729	0.893941	2.43135	0.00735	0.583218
38	Erp27	0.214238	1.4716	2.7801	0.02635	0.894542
39	F730043M19Rik	0.098873	0.764374	2.95064	0.04555	0.999566
40	Fam126b	0.082416	0.527242	2.67747	0.00835	0.62567
41	Fam199x	0.086256	0.549261	2.6708	0.0222	0.855025
42	Fam208a	0.82376	4.34782	2.4	0.02595	0.892352
43	Fancm	0.164516	0.786702	2.25759	0.02405	0.87935
44	Fbxl3	2.55853	12.3287	2.26863	0.0018	0.393035
45	Firre	0.128454	0.977672	2.92809	0.01045	0.66754
46	Fktn	0.084023	0.503534	2.58323	0.0101	0.652143
47	Flt1	0.133853	0.735772	2.45861	0.0366	0.999566
48	Fmo1	0.412602	2.52944	2.616	0.0014	0.36974
49	G2e3	0.295723	1.86296	2.65527	0.0277	0.90476
50	Gad1	0.258822	1.08118	2.06257	0.0375	0.999566
51	Gcnt1	0.759003	3.31569	2.12713	0.0122	0.692519
52	Alb	1.23843	0.07599	-4.02655	0.03025	0.935336
53	Arhgap22	0.799771	0.085624	-3.22349	0.01465	0.759724
54	Ece1	20.1341	3.86064	-2.38273	0.0022	0.426593

Table S4: Mouse or rat immunization regime to raise antibodies against *Pb*TIP

Day	Dose of *Pb*TIP Mouse/rat	Schedule
1st	30µg/50µg	Priming
15th	20µg/ 30µg	1st boost
21st	20µg/ 30µg	2nd boost
28th	20µg/ 30µg	3rd boost

Supplementary figures

Figure S1

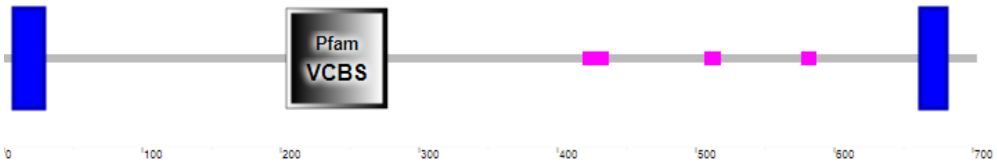

Figure S2

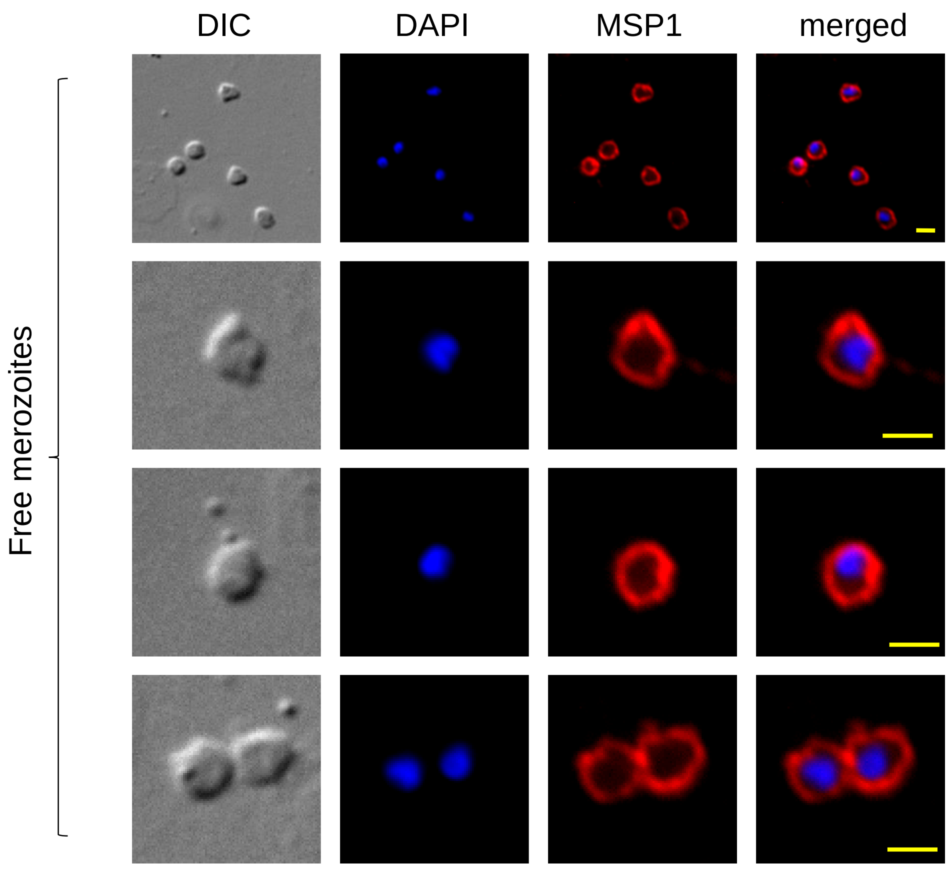

Figure S3

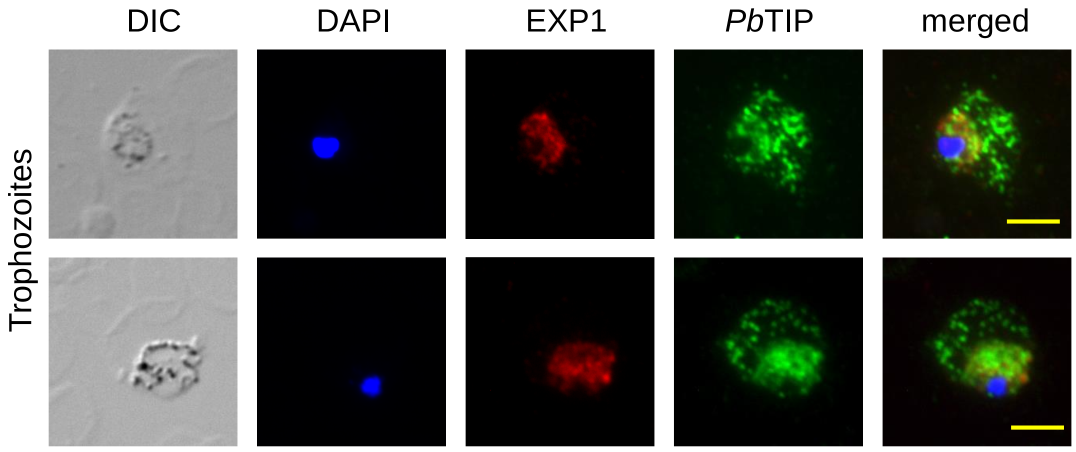

Figure S4

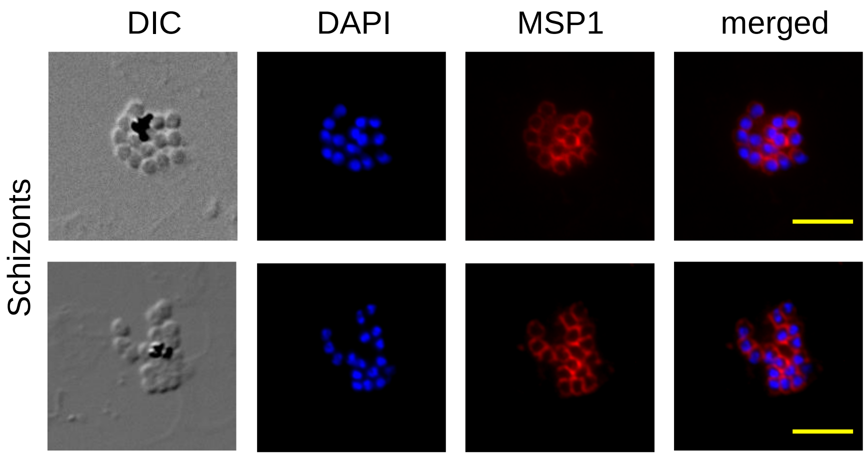

Figure S5

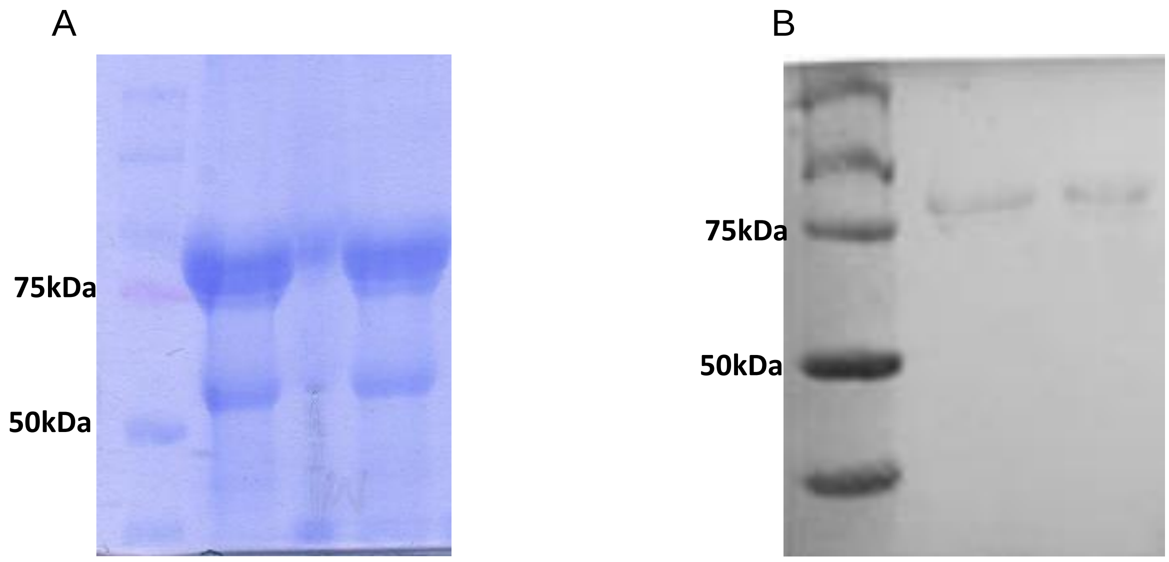

Figure S6

```
PbTIP     MVKCGKYVVILELLLLTLLYNLIKRISISNNGEIIPSFVDAYNWNILERWKAITPKEKLE     60
TF55      ------------------------------------------------------------     0

PbTIP     YKVNHDLGLDIDAEIGDFGDYNSDVKTDLILFKYDKTTMTSTIFVYIFSPSENKFVYHTE     120
TF55      ------------------------------------------------------------     0

PbTIP     AKLEGKIVNLMVIDLNFDGSLDVLVLFKDNNSQDKFYISTFIQNESDELEERFNSKIKET     180
TF55      --------NLMVIDLNF-------------------------------------------     9
                  *********

PbTIP     ENESISDLEDQSFYFTNIHPLLCDINNDGLPDLIAQHPNKSKSFSRFIWINNGNDGFKSI     240
TF55      ------------------------------------------------------------     9

PbTIP     LWENMDLFEYTDLSEISNPNSNAIVDLNGDCKADLVFTVINKKNTKRIFLEIWINKIVNG     300
TF55      ------------------------------------------------------------     9

PbTIP     KSRYVKADEDYMLPANSMQIIFADFNADGSIDMVVPTCVKSSSCNYCCTRGDKIYFIPNI     360
TF55      ------------------------------------------------------------     9
```

Figure S7

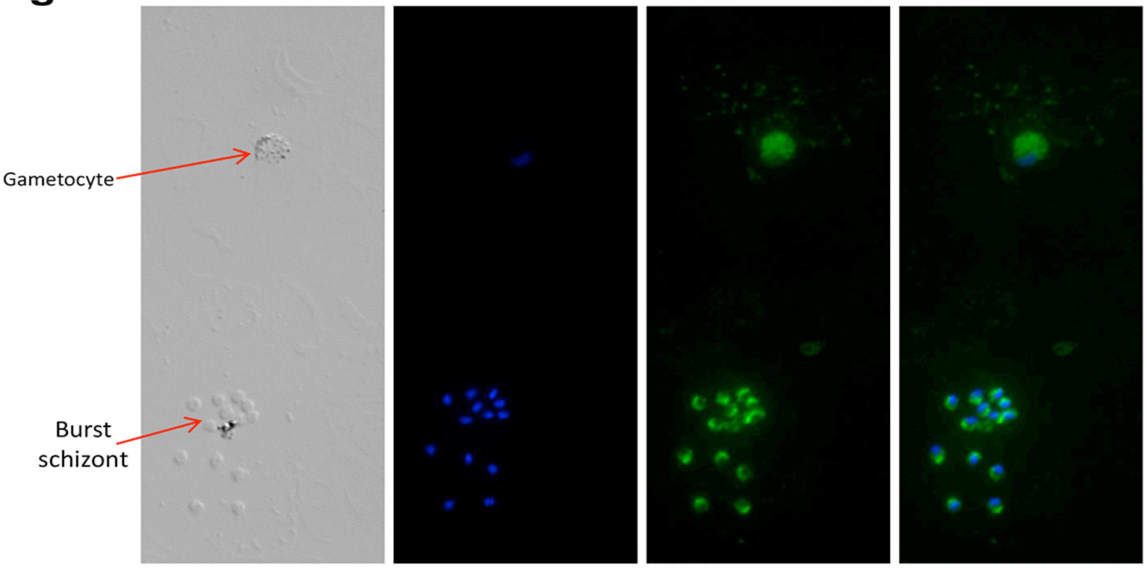

Figure S8

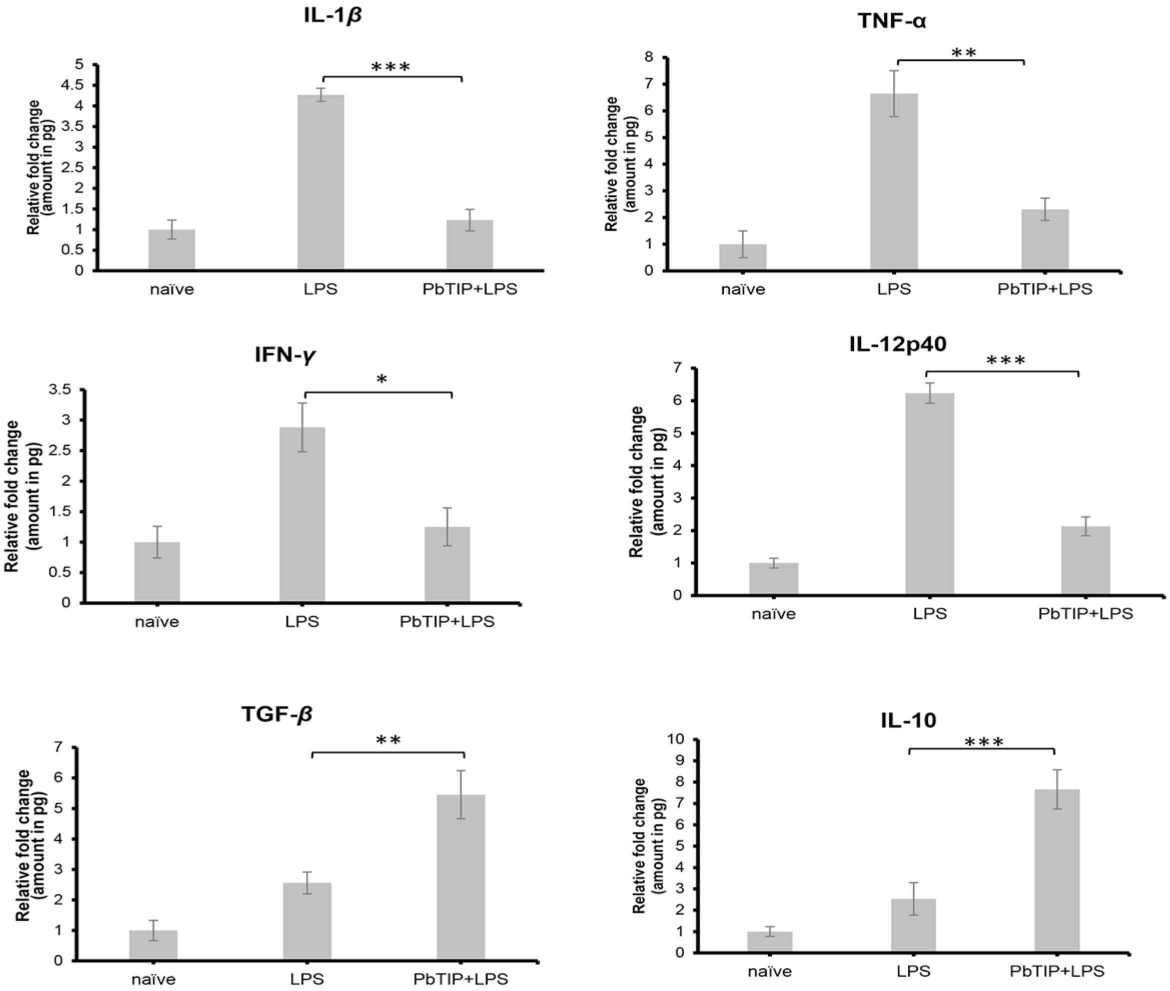

Figure S9

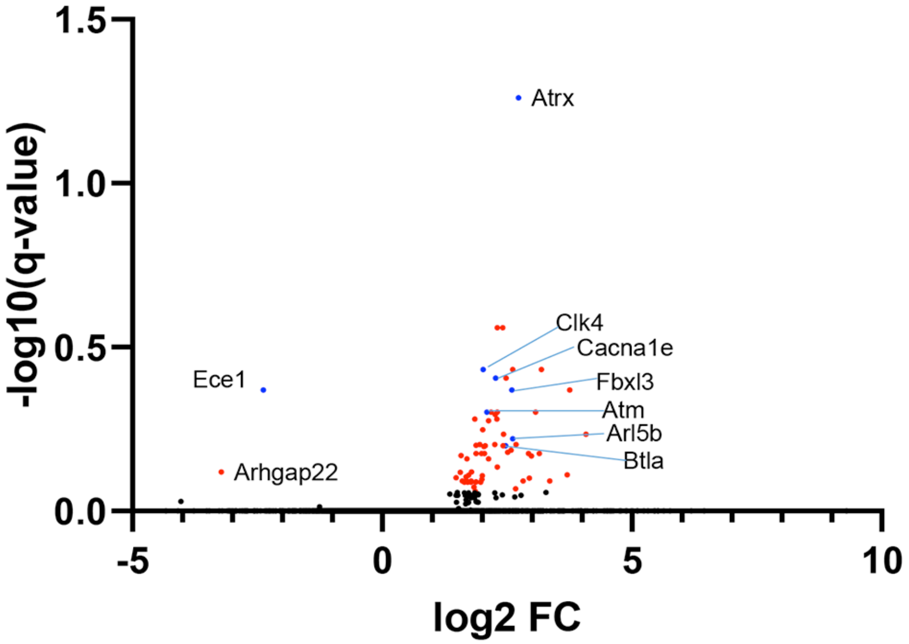

Figure S10

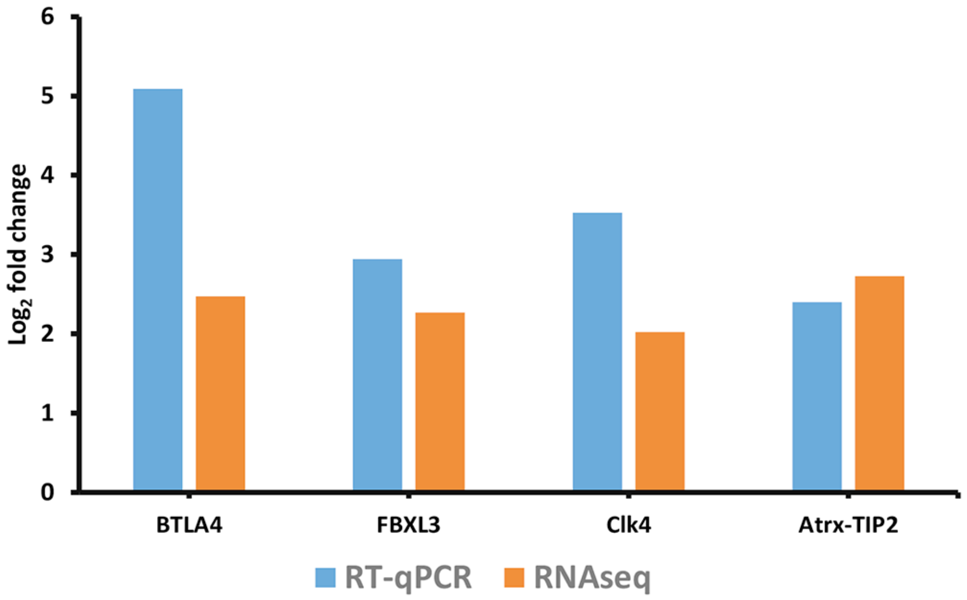

Figure S11

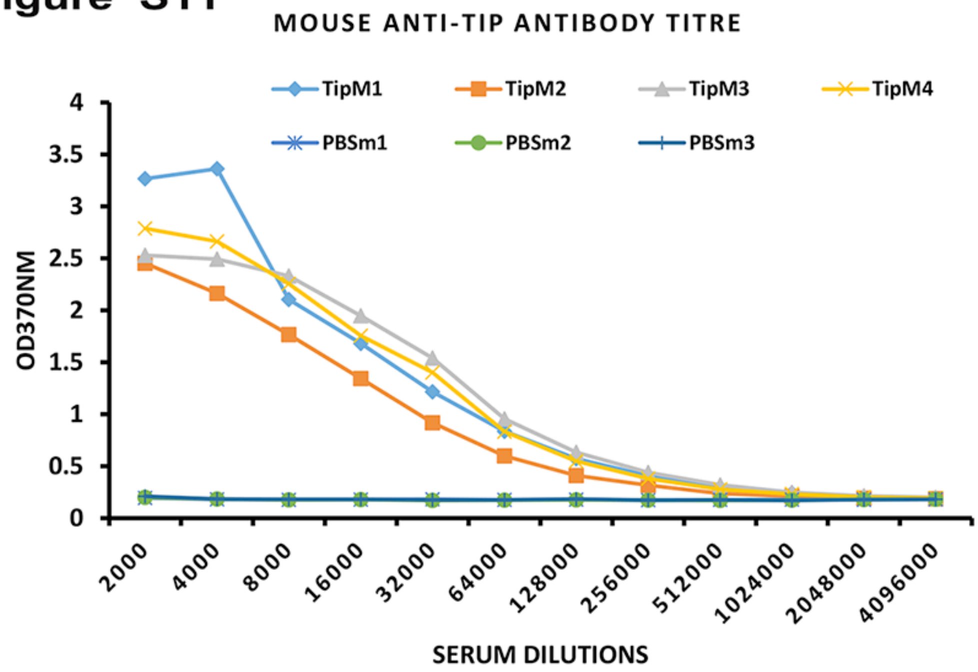
MOUSE ANTI-TIP ANTIBODY TITRE
TipM1
TipM2
TipM3
TipM4
PBSm1
PBSm2
PBSm3
OD370NM
4
3.5
3
2.5
2
1.5
1
0.5
0
2000
4000
8000
16000
32000
64000
128000
256000
512000
1024000
2048000
4096000
SERUM DILUTIONS
